# Type I-Fv and engineered type IV-A1 CRISPR–Cas effectors facilitate genome reduction in *Escherichia coli*

**DOI:** 10.1093/nar/gkaf1399

**Published:** 2025-12-18

**Authors:** Nathalie Klein, Mariana Sanchez-Londono, Meral M Kara, José Vicente Gomes-Filho, Samuel Novak, Karim H Kholeif, Lukas Pekarek, Neva Caliskan, Lennart Randau

**Affiliations:** Department of Biology, Philipps-Universität Marburg, Hans-Meerwein-Straße 6, 35043 Marburg, Germany; Department of Biology, Philipps-Universität Marburg, Hans-Meerwein-Straße 6, 35043 Marburg, Germany; Department of Biology, Philipps-Universität Marburg, Hans-Meerwein-Straße 6, 35043 Marburg, Germany; Department of Biology, Philipps-Universität Marburg, Hans-Meerwein-Straße 6, 35043 Marburg, Germany; Helmholtz Institute for RNA-based Infection Research (HIRI-HZI), Josef-Schneider-Straße 2, 97080 Würzburg, Germany; Department of Biology, Philipps-Universität Marburg, Hans-Meerwein-Straße 6, 35043 Marburg, Germany; Helmholtz Institute for RNA-based Infection Research (HIRI-HZI), Josef-Schneider-Straße 2, 97080 Würzburg, Germany; Helmholtz Institute for RNA-based Infection Research (HIRI-HZI), Josef-Schneider-Straße 2, 97080 Würzburg, Germany; Department of Biochemistry III, University of Regensburg, Universitätsstraße 31, 93053 Regensburg, Germany; Department of Biology, Philipps-Universität Marburg, Hans-Meerwein-Straße 6, 35043 Marburg, Germany; Center for Synthetic Microbiology (SYNMIKRO), Karl-von-Frisch-Straße 14, 35043 Marburg, Germany

## Abstract

Class 1 CRISPR–Cas systems utilize multi-subunit effector ribonucleoprotein complexes to identify and target DNA. Upon recognition, type I systems recruit the helicase/nuclease Cas3 for DNA degradation, while type IV-A systems use the helicase CasDinG for transcriptional repression. Here, we developed two recombinant class 1 CRISPR–Cas genome editing tools for inducing large genomic deletions: the compact type I-Fv (also termed I-F2) system from *Shewanella putrefaciens* and the type IV-A1 system from *Pseudomonas oleovorans*. In the latter, CasDinG was engineered to include a C-terminal HNH nuclease domain, conferring DNA cleavage activity and enabling analysis of CasDinG processivity. Whole-genome sequencing of *Escherichia coli* BL21-AI was used to monitor genome reduction and DNA repair mechanisms in response to CRISPR–Cas-induced damage. Small deletions were flanked by microhomologies, consistent with repair via alternative end joining, whereas deletions larger than 10 kb consistently terminated at nearby IS1 elements, implicating these sequences in the repair process. This study introduces compact type I and engineered type IV-A genome editing tools with distinct protospacer-adjacent motif requirements and provides new insights into CasDinG evolution and the DNA repair pathways engaged during CRISPR–Cas-mediated genome editing.

## Graphical Abstract

**Graphical Abstract:**
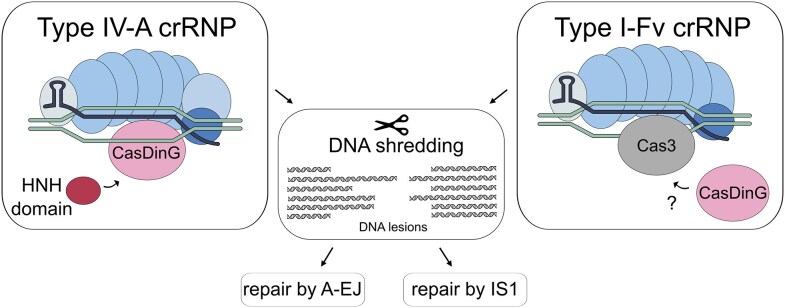

## Introduction

Clustered regularly interspaced short palindromic repeats (CRISPR) and CRISPR-associated (Cas) genes provide prokaryotes an adaptive immune system that enables defense against mobile genetic elements (MGEs), such as viruses or plasmids [1]. CRISPR RNAs (crRNAs) assemble with Cas effector proteins and direct them to complementary target sequences, leading to the nucleolytic degradation of the targets [2]. Based on their protein composition, these highly diverse systems are classified into class 1 systems, which use multiprotein effector complexes, and class 2 systems, which utilize single multidomain effectors for target interference [3].

Beyond their natural function, CRISPR–Cas effectors—particularly class 2 systems with Cas9—are widely used for gene-editing applications, often by introducing double-strand breaks into target DNA [4]. Class 1 systems, especially type I, are increasingly being adapted for genome editing [5, 6], transcriptional regulation [7, 8], long-range genomic deletions [9], genomic integration [10], and base editing [8, 11].

Type IV systems, a subgroup of class 1, are known to function in the clearance of MGEs—particularly conjugative plasmids—and in gene regulation, without requiring a nuclease [12–15]. These systems are highly diverse and possess unique features that complicate the tracing of their evolutionary history [13]. The type IV-A1 system of *Pseudomonas oleovorans* has been shown to regulate the transcript levels of the host gene *pilN* via a self-targeting spacer, representing a natural CRISPR interference (CRISPRi) mechanism. Recently, it was demonstrated that, compared to the widely used dCas9 CRISPRi system, type IV-A can downregulate genes more effectively and over a broader range [16].

The multieffector complex of IV-A systems is composed of (i) several subunits of Cas7 that define the crescent effector backbone, (ii) the endoribonuclease Cas6 responsible for pre-crRNA processing, and (iii) Cas5 and Cas8 forming the protospacer-adjacent motif (PAM) recognition interface of the complex [17–19]. The type IV-A interference mechanism relies on PAM-dependent target recognition (5′-AAG-3′) and binding by the effector complex followed by the recruitment of the CRISPR-associated DinG (CasDinG) [18, 19]. The ATP-dependent 5′-3′ DNA translocation of the helicase CasDinG results in transcriptional inhibition of the targeted region [20, 19]. This activity of CasDinG is only dispensable if the effector directly targets the promoter region of the repressed gene [21, 22, 16].

This recruitment mechanism parallels that of Cas3 in type I CRISPR–Cas systems. Here, the multiprotein effector complex Cascade (CRISPR-associated complex for antiviral defense) processes pre-crRNAs, recognizes and binds target DNA, and facilitates R-loop formation [2, 23, 24]. Type I effectors typically form a seahorse-shaped structure consisting of Cas6 bound to the 3′-end of the crRNA, several Cas7 subunits forming the backbone, Cas11 forming the “belly,” and Cas5 and Cas8 at the 5′-end [25, 26, 6]. Cas3 is recruited to Cascade, where it mediates progressive uni- or bidirectional degradation of target DNA through its HD-nuclease and ATP-dependent helicase domains [27]. Type I CRISPR–Cas systems are diverse and comprise several subtypes (I-A to I-G) [28]. The type I-Fv (also termed I-F2) system from *Shewanella putrefaciens* represents a minimal CRISPR–Cas system and its Cascade complex recognizes a 5′-GG-3′ PAM and comprises only three Cas proteins (Cas6, Cas7, and Cas5) and it recruits a Cas2/3 fusion protein for interference [29, 30]. Unusually, this special system also includes a CasDinG protein, which is distinct from the chromosomally encoded DinG involved in DNA repair [29, 31]. CasDinG was found to be nonessential in conjugation assays [29], and its possible function within type I mediated immunity remains unclear. In addition, the directionality of the type I-Fv system of *S. putrefaciens* remained unknown.

In contrast, CasDinG in type IV-A systems is better characterized. It contains two superfamily 2 helicase domains and accessory domains such as an arch domain, residual Fe-S clusters, and an N-terminal domain (NTD) [20, 18, 19]. CasDinG can unwind dsDNA and RNA/DNA hybrids in a 5′-3′ direction, and its Walker A and B motifs facilitate ATP binding and hydrolysis [20, 21, 15]. Nevertheless, its processivity *in vivo* remains uncharacterized. Recently, a type IV-A system in *Sulfitobacter* sp. JL08 was identified in which CasDinG contains an additional C-terminal HNH-nuclease domain capable of specific DNA cleavage [32]. The HNH domain likely mediates cleavage via a mechanism similar to that of Cas3, combining unwinding with processive DNA degradation.

In this study, we engineered a fusion of the HNH domain from *Sulfitobacter* sp. JL08 CasDinG to the CasDinG from *P. oleovorans* (*Po*CasDinG), generating a type IV-A1 CRISPR–Cas system with *in vivo* DNA cleavage activity. We also investigated genome editing using the minimal type I-Fv system from *S. putrefaciens*, with its associated CasDinG protein and a K82A Walker A motif mutant. Using whole-genome sequencing of *Escherichia coli* BL21-AI, we analyzed the extent and characteristics of genome deletions induced by both systems and uncovered distinct DNA repair mechanisms, including microhomology-mediated repair and insertion sequence (IS1)-associated resolution of large deletions. Our findings provide both practical genome editing tools and mechanistic insights into CRISPR-associated DNA repair pathways.

## Materials and methods

### Single molecule measurements of CasDinG recruitment

For protein production, a pET20b(+) plasmid encoding *casding* with a C-terminal His_6_-Tag [19] was transformed into *E. coli* BL21-AI and plated on LB-agar plates containing ampicillin (120 µg/ml). Overnight cultures were used to inoculate 250 ml of LB-media supplemented with ampicillin (100 µg/ml) (LB-Amp) to an OD_600_ of 0.1. The cells were incubated shaking at 37°C until reaching an OD_600_ between 0.5 and 0.6. Gene expression was induced with 0.25 mM isopropyl-β-d-thiogalactopyranoside (IPTG) and 0.2% arabinose. Cells were grown overnight at 18°C and harvested by centrifugation at 8000 × *g* for 20 min at 4°C. Five to ten milliliters of lysis buffer [50 mM Tris, pH 8 (at 4°C), 20 mM Imidazole, 1 M NaCl, 10% glycerol] was used to dissolve the pellet prior to lysis. Cells were lysed by sonication at 40% amplitude with pulses. The lysate was clarified by centrifugation (14 000 × *g*, 40 min) followed by filtration (0.22-µm filter) and loaded to a 1 ml HisTrapHP column (Cytiva) pre-equilibrated with buffer A (50 mM Tris, pH 8, 20 mM Imidazole, 300 mM NaCl, 10% Glycerol) at 4°C. The column was washed with 10 column volumes of buffer A. Bound CasDinG was eluted with a gradient of buffer B (50 mM Tris, pH 8, 500 mM Imidazole, 300 mM NaCl, 10% Glycerol). Peak fractions were further purified by size exclusion chromatography using a Superose 6 Increase 10/300 GL column (Cytiva) equilibrated with size exclusion buffer (50 mM Tris, pH 8, 300 mM NaCl) at 4°C.

To express type IV-A1 effector complexes without CasDinG, previously described vectors [19] were co-transformed into *E. coli* BL21-AI and plated on LB-agar containing kanamycin (50 µg/ml) and ampicillin (100 µg/ml). Expression and purification were performed as previously described for type IV-A1 effector complexes with CasDinG [19]. Protein concentrations were determined by absorbance at 280 nm using a NanoPhotometer NP80 (IMPLEN), with extinction coefficients calculated based on a 1:1:5:1 stoichiometry of Cas5:Cas8:Cas7:Cas6.

CasDinG was labeled with an NHS-reactive dye using the Monolith Protein Labeling Kit RED-NHS (NanoTemper Technologies), which targets primary amines (e.g. lysine residues), forming covalent bonds. DNA handles for optical tweezer experiments were generated using a 13.4-kb λ-DNA vector as a polymerase chain reaction (PCR) template. A 32-nt target sequence containing a PAM was inserted using Golden Gate assembly with BsaI-HFv2, resulting in an 11.4-kb DNA construct. DNA handles were gel-purified, and both ends were biotinylated using Biotin-16-dUTP. The final product was purified using a PCR cleanup kit (Macherey-Nagel).

Measurements were performed using a commercial confocal-assisted optical tweezers system (C-Trap, LUMICKS). Streptavidin-coated polystyrene beads were used to tether the biotinylated DNA handles. The beads and DNA were introduced into a five-channel microfluidic flow cell. Tether formation was confirmed using force–extension (F–x) curves. The tethered DNA was then exposed to fluorescently labeled CasDinG and purified type IV-A1 effector complexes in subsequent channels. Fluorescence data were collected using confocal microscopy.

All data acquisition was performed using Bluelake software. Files were saved as .h5 format for analysis using Python scripts. F–x curves were analyzed using the POTATO (Practical Optical Tweezers Analysis Tool) script [33]. Significant events were analyzed in Fiji (ImageJ), and fluorescent spot tracking was performed using the TrackMate plugin. The distance fraction (θ) between beads was calculated to determine the protein binding position on DNA, with a target θ value of 0.564. Frames were oriented consistently to maintain this reference point. A total of 224 frames from three videos were analyzed, with two datasets: one including frames with DNA stretching (*n* = 224) and one excluding them (*n* = 191).

### Structure prediction and comparison

The structure of engineered CasDinG from *P. oleovorans*, containing the C-terminal HNH domain from *Sulfitobacter* sp. JL08, was predicted using AlphaFold2 via the ColabFold server [34, 35]. The predicted model was aligned with the published cryogenic electron microscopy (cryo-EM) structure of CasDinG (PDB: 8RFJ) using ChimeraX-1.9 [36].

### Cloning of constructs

The HNH domain sequence from *Sulfitobacter* sp. JL08 was amplified from the vector pHS1184 (Addgene plasmid #205965). The gel-purified fragment was cloned via Gibson assembly [37] into a plasmid encoding the type IV-A1 *cas* genes (pSR13) constructed in [38] or a plasmid encoding *cas* genes with *casdinG*, where the NTD was deleted by inverse PCR (pNK103). The HNH domain was also cloned into plasmid pSR77, which carries all *P. oleovorans* type IV CRISPR–Cas components and a repeat-randomized spacer-repeat cassette to generate an all-in-one solution.

For type I-Fv constructs, a pCDF-Duet™-1 plasmid was used to insert a minimal CRISPR-array containing spacer sequences with two type IIS restrictions sites enabling exchange of these sequences with Golden Gate cloning [39]. Therefore, a pCDF-Duet™-1 plasmid was digested with NotI and BamHI (New England BioLabs) and four phosphorylated complementary oligonucleotides were annealed and ligated into the vector, yielding pMK1.1. CasDinG was PCR-amplified from *S. putrefaciens* genomic DNA and inserted into pMK1.1 via Gibson assembly, resulting in plasmid pMK1. The spacer sequence was exchanged to target *lacZ* using BseRI and oligonucleotides with compatible overhangs, generating pMK3. A site-directed mutation (K82A) in pMK1 was introduced to inactivate CasDinG’s ATPase domain, resulting in plasmid pMK4. Using Golden Gate cloning, the spacer sequence has been exchanged to spacers targeting *lacZ* (pMK5) or *dmpG* (pMK25, pMK26, pMK31, pMK32). All plasmids were transformed into chemically competent *E. coli* DH5α, and sequences were verified via Sanger sequencing. Lists of plasmids (Supplementary Table S2), protospacer sequences (Supplementary Table S3), and Genbank files are provided in the Supplementary data.

### Repression and deletion assay in *E. coli* BL21-AI

*Escherichia coli* BL21-AI cells were co-transformed with either a one-plasmid system, encoding type IV-A1 constructs targeting *lacZ* (pMSL89) or the tRNA-Asp region (pLCB1), or a two-plasmid system in which one encoded the respective *cas* genes (type IV-A1 or type I-Fv), and the other contained a CRISPR array targeting either *lacZ* or *dmpG*. Selection plates were supplemented with kanamycin (50 µg/ml) for the one-plasmid system, or with spectinomycin (100 µg/ml) and ampicillin (120 µg/ml) for two-plasmid type IV-A1 constructs, or spectinomycin (100 µg/ml) and kanamycin (50 µg/ml) for type I-Fv constructs. After overnight growth at 37°C, colonies were inoculated into 3 ml LB with the corresponding antibiotics and grown overnight. Cultures were adjusted to an OD_600_ of 1. A 10-fold serial dilution (10^−1^ to 10^−5^) was plated on induction plates containing 0.2% arabinose, 1 mM IPTG, X-Gal (40 μg/ml), and the appropriate antibiotics. Plates were incubated at 37°C for 16–24 h and stored at 4°C for 2 days before colony color assessment. Blue-to-white colony ratios were calculated. Nontargeting CRISPR array plasmids served as controls.

### Primer walking PCR

To verify genome editing and identify deletion boundaries, colonies lacking *lacZ* expression (white colonies) were subjected to colony PCR using DreamTaq Master Mix (Thermo Fisher). Genes located up- and downstream of the target site were screened using a primer walking strategy (primer sequences are detailed in Supplementary Table S4). When amplification indicated the presence of flanking regions, PCR products spanning the predicted junction sites were sequenced by Sanger sequencing.

### Genomic DNA extraction from engineered *E. coli*

Two sequencing rounds were performed. In the first round, 200 colonies derived from 2 independent transformations were selected per assay. In the second round, 90 colonies originating from 30 independent transformations were collected to increase the heterogeneity of the deletion range. Colonies were inoculated in 200 µl LB medium in 96-well plates, and grown overnight at 37°C with shaking. Optical density at 600 nm (OD_600_) was measured, and cultures were pooled to a final OD_600_ of 1. Genomic DNA was extracted using the NucleoSpin Tissue Kit (Macherey-Nagel) following the bacterial protocol, with elution in nuclease-free water. DNA concentrations were measured using a Qubit fluorometer, and purity was assessed via A260/280 ratios using a NanoPhotometer NP80 (IMPLEN). Samples were submitted to Novogene for Illumina whole-genome sequencing (see Supplementary Table S5 for details).

### Identification of deletion boundaries from Illumina WGS

Whole-genome sequencing (WGS) data were subjected to quality control using FastQC (v.012.0) [40]. Potential adapter contamination was identified and removed with Cutadapt v.5.0 [41]. Prior to alignment, a reference sequence file was generated, containing the *E. coli* Bl21-AI genome and all plasmids utilized in the experimental setup. To identify genomic deletions, reads were aligned to this custom reference using HISAT2 v.2.2.1 [42], allowing for the reporting of spliced reads. Subsequently, BAM files were loaded into R and processed using a suite of packages. Specifically, Gviz v1.52.0 [43], Rsamtools v2.24.0 [44], and GenomicRanges v1.6.0 [45] were utilized to identify and summarize spliced reads within the predefined target region. Junction-spanning reads (CIGAR containing “N”) with mapping quality score MAPQ ≥60 were then extracted from the BAMs using a custom shell script (splice_extractor.sh). Junctions were subsequently called and analyzed with a companion Python script (detect_junctions.py), which (i) collapses near-identical junctions within a user-defined tolerance, (ii) restricts calls to specified genomic intervals, and (iii) annotates each junction with overlapping features (e.g. genes or insertion sequences) from a supplied annotation file. Unless stated otherwise, parameters were --tol 3 and --flank 50. All shell and R scripts generated for this analysis are publicly available at https://github.com/VicenteBR/Deletion-boundary-detection--Klein-et-al--2025-.

## Results

### CasDinG is recruited to target bound type IV-A1 effector complexes

**Figure 1.**
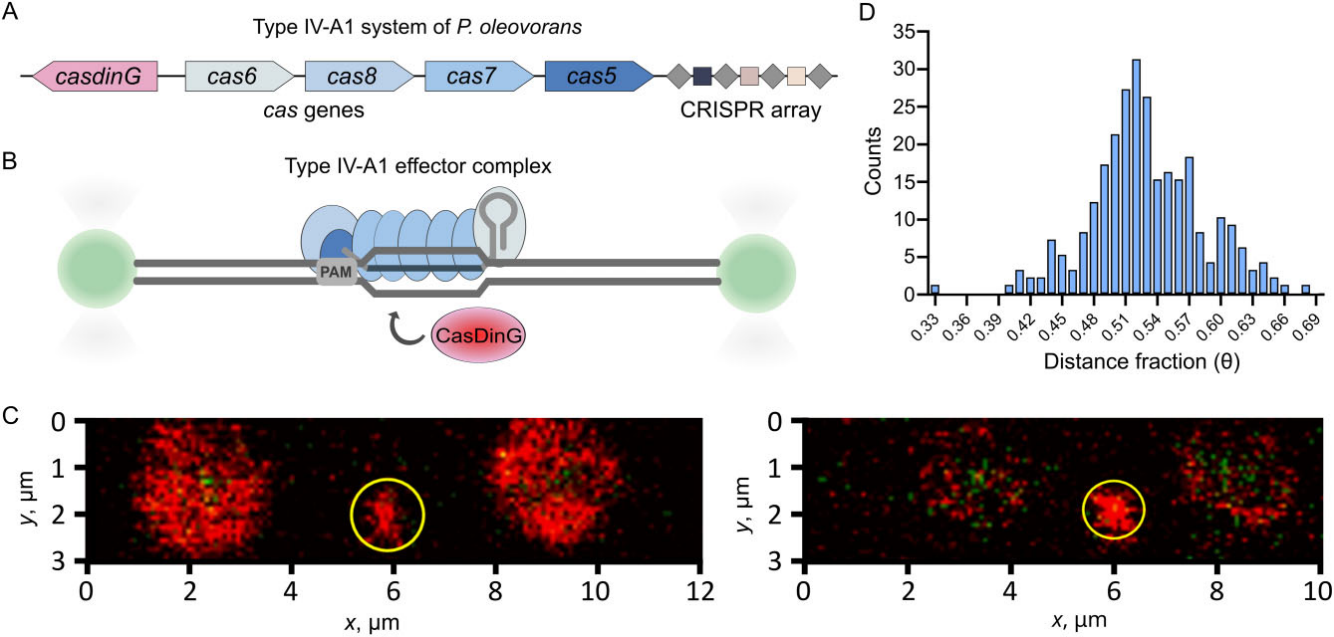
Visualization of CasDinG recruitment. (**A**) Schematic representation of the type IV-A1 operon of *P. oleovorans* comprising a CRISPR array, casdinG, cas6, cas8, cas7, and cas5. (**B**) Schematic setup of the C-trap measurements. Double-stranded DNA is tethered between two beads. A target sequence with a flanking PAM sequence allows binding of the effector complex and recruitment of CasDinG. (**C**) Snapshots of measurements during confocal microscopy. Yellow circled is the red signal of lysine-labeled CasDinG at the target DNA. The beads’ auto emission signal is shown in green. Aggregates of CasDinG were bound to the beads emitting red signals. *x* and *y* axes shows distances in µm. (**D**) Histogram of protein signal counts per position for CasDinG measurements Pearson’s normality test significance level α = 0.05.

Type IV-A complexes require recruitment of the ATP-dependent helicase CasDinG to mediate interference [18, 19]. To visualize this recruitment, we combined purified *P. oleovorans* type IV-A1 effector complexes and *Po*CasDinG with a confocal-assisted optical tweezers system (C-Trap, LUMICKS) integrated with microfluidics and fluorescence microscopy (Fig. 1A and B). We inserted a target sequence flanked by a PAM into a 13.4-kb λ-phage DNA plasmid and amplified the DNA by PCR. The DNA tether was immobilized between two optical traps and sequentially exposed to type IV-A1 complexes and fluorescently labeled CasDinG using a five-channel microfluidics setup. Fluorescence microscopy enabled the visualization of CasDinG recruitment to its target site (Fig. 1B).

We observed signals of lysine-labeled CasDinG in three independent measurements showing its recruitment to the target DNA (Fig. 1C and Supplementary Video S1). All frames were pooled and the distance fractions (*θ)*, indicating the positions of the signal between the beads, were analyzed. Due to random binding of the DNA tether to the beads, the DNA can adopt two different orientations. As the target site is located at position *θ* = 0.558, each dataset was aligned accordingly. We measured mean distance fractions of protein signals at *θ* = 0.530 (Fig. 1D). This slight difference may result from CasDinG activity, as it might begin translocating shortly after recruitment. A total of 326 PAM sequences flanked by random sequences all over the DNA tether as well as measurements lacking type IV-A1 complexes served as negative controls (Supplementary Fig. S1). In none of the measurements, CasDinG signals were observed confirming the specific recruitment of CasDinG to type IV-A1 effector bound target DNA.

### Engineering a DNA-cleaving variant of the type IV-A1 CRISPR–Cas system of *P. oleovorans*

The type IV-A CRISPR–Cas system of *Sulfitobacter* sp. JL08 encodes a CasDinG containing an HNH-nuclease domain and was reported to cleave target DNA [32]. CasDinG–HNH moves in 5′-3′ direction along the nontargeting DNA strand that was shown to result in continuous cleavage of both strands [32].

**Figure 2.**
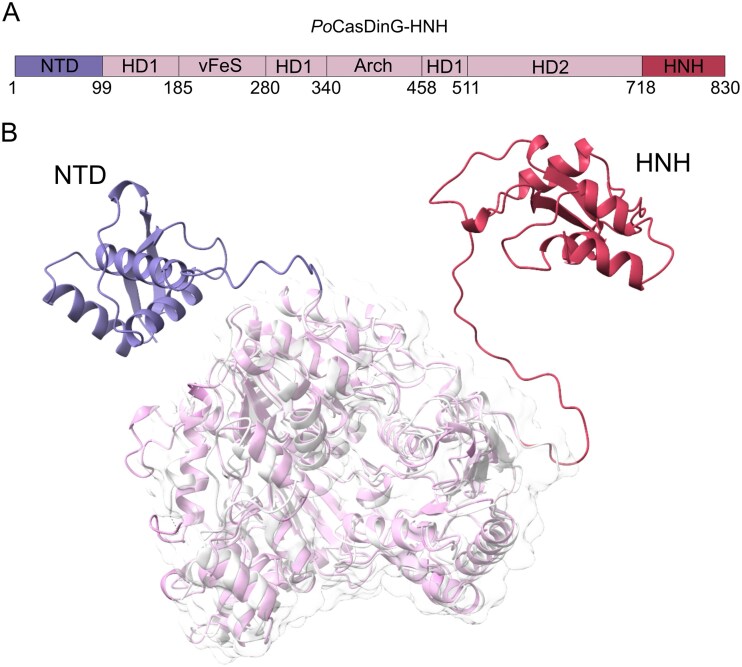
Structure prediction of engineered PoCasDinG. (**A**) Schematic presentation showing protein domain organization of engineered PoCasDinG. (**B**) CasDinG helicase core (light pink), NTD (purple), and HNH (red) were predicted by AlphaFold 2 and aligned to the cryoEM structure PoCasDinG which is missing the NTD (PDB:8RFJ; white).

Here, we fused the HNH nuclease domain of CasDinG found in *Sulfitobacter* sp. JL08 to the C-terminus of *Po*CasDinG that does not exhibit natural DNase activity (Fig. 1A). We predicted the structure of the engineered *Po*CasDinG–HNH using AlphaFold2 program on the ColabFold server and aligned the predicted structure to the cryo-EM structure of the native *Po*CasDinG (Fig. 2). Both structures align well with a root mean square deviation of 1.623 Å. Both the NTD and the HNH domain appeared as flexible regions with long linkers, suggesting conformational variability upon recruitment. To further assess the role of the NTD, we also created a CasDinG–HNH variant lacking this domain.

### HNH fusion enables DNA cleavage by the type IV-A1 CRISPR–Cas system

**Figure 3.**
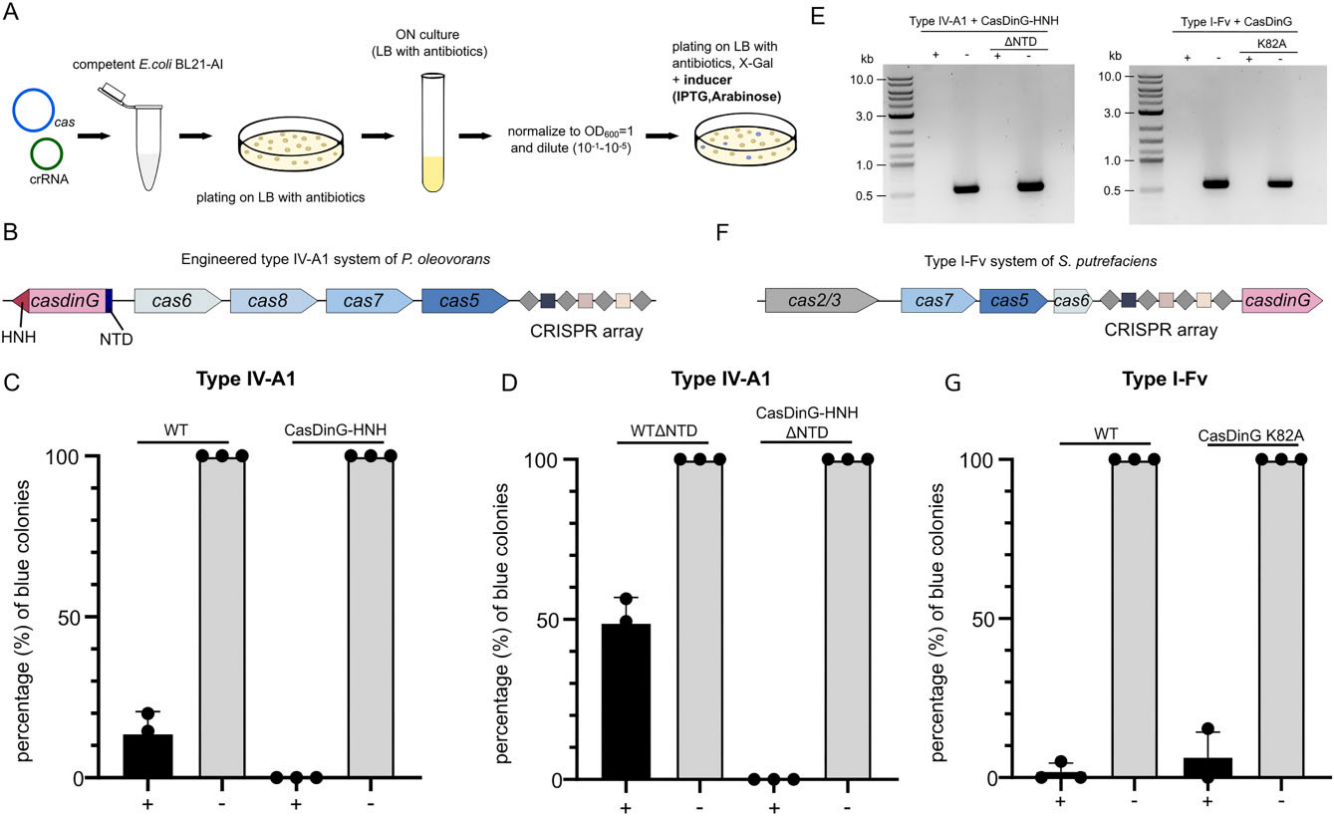
Genomic targeting with engineered type IV-A1 and type I-Fv CRISPR–Cas systems. (**A**) Schematic of workflow used to test the engineered variants of type IV-A1 and the type I-Fv system in *E. coli* BL21-AI cells by targeting *lacZ*. (**B**) Engineered type IV-A1 CRISPR–Cas locus of *P. oleovorans*. (**C**) Percentage of blue colonies after genomic targeting assay of wild-type (WT) type IV-A1 and the engineered variants comprising the HNH-domain (CasDinG–HNH) for targeting (+) and nontargeting (−) samples. (**D**) Same as in panel (C), but with engineered construct lacking the NTD. (**E**) Agarose gels of *lacZ* PCR verifying the absence of the gene in the genomes assay with a targeting spacer (+, white colonies) in contrast to the nontargeting control (−, blue colonies). (**F**) Type I-Fv CRISPR–Cas locus of *S. putrefaciens*. (**G**) Same as in panels (C) and (D), but with type I-Fv with CasDinG (WT) and a CasDinG mutant (CasDinG K82A). *t*-test: type IV: WT (*P* < .0001), WTΔNTD (*P* < .0004); no *t*-test for CasDinG–HNH or CasDinG–HNHΔNTD (0% blue colonies). type I-Fv: WT and CasDinG K82A (*p* < .0001).

We utilized *lacZ* deletion assays [9, 15] in *E. coli* BL21-AI to test whether the engineered CasDinG–HNH variants could induce DNA cleavage. Cells were transformed with plasmids encoding a minimal CRISPR array targeting *lacZ*, type IV-A1 effector proteins, and either the CasDinG–HNH fusion or the ΔNTD variant (Fig. 3A and B). As controls, native type IV-A1 complexes with a *lacZ* spacer and nontargeting controls containing a random spacer sequence were included. On X-Gal-containing inducer plates, the presence of white colonies indicated either *lacZ* repression or gene deletion. Control samples with native *Po*CasDinG (without nuclease activity) showed an 87% reduction of blue colonies between targeting and nontargeting conditions (Fig. 3C). The engineered CasDinG–HNH and CasDinG–HNHΔNTD both yielded only white colonies (Fig. 3C and D), suggesting that the expected DNA deletions may enhance the *lacZ* transcriptional-repression phenotype.

In this case, PCR analysis confirmed the absence of the *lacZ* gene in white colonies (Fig. 3E). Both engineered variants efficiently deleted *lacZ*, indicating that the HNH domain conferred DNA cleavage activity. The ΔNTD variant was equally efficient, suggesting that the NTD is dispensable for cleavage. However, in the native system (without HNH), deletion of the NTD impaired but did not abolish interference activity, as evidenced by an increased proportion (48% over 13%) of blue colonies. Thus, while the NTD contributes to CRISPRi efficiency, it is not essential. Together, these results demonstrate that fusing an HNH domain to *Po*CasDinG creates a functional type IV-A1 system capable of targeted DNA degradation.

We aimed to compare type I- and type IV-mediated genome deletion activities and optimized the type compact I-Fv CRISPR–Cas system of *S. putrefaciens* for plasmid-based genome engineering. This system contains a three-protein effector complex without Cas8 and has been described to interfere with phages and plasmids [29, 30]. Here, we used the type I-Fv effector complex with Cas2/3 and CasDinG to target *lacZ* in the genome of *E. coli* BL21-AI (Fig. 3E).

To assess the role of CasDinG, we tested both the wild-type type I-Fv system and a mutant version containing an ATPase-inactive CasDinG (K82A) targeting *lacZ*. The deletion efficiency was evaluated using the blue/white colony assay described earlier (Fig. 3A). Both the wild-type and mutant systems showed mostly white colonies (Fig. 3G), and PCR confirmed that *lacZ* was deleted in both cases (Fig. 3E). These results indicate that the type I-Fv system is capable of mediating targeted DNA degradation in *E. coli*, and that CasDinG is not essential for this activity. However, its presence may influence the size or efficiency of the deletions, as explored further below.

### Engineered type IV-A1 and type I-fv systems induce large genomic deletions in *E. coli*

**Figure 4.**
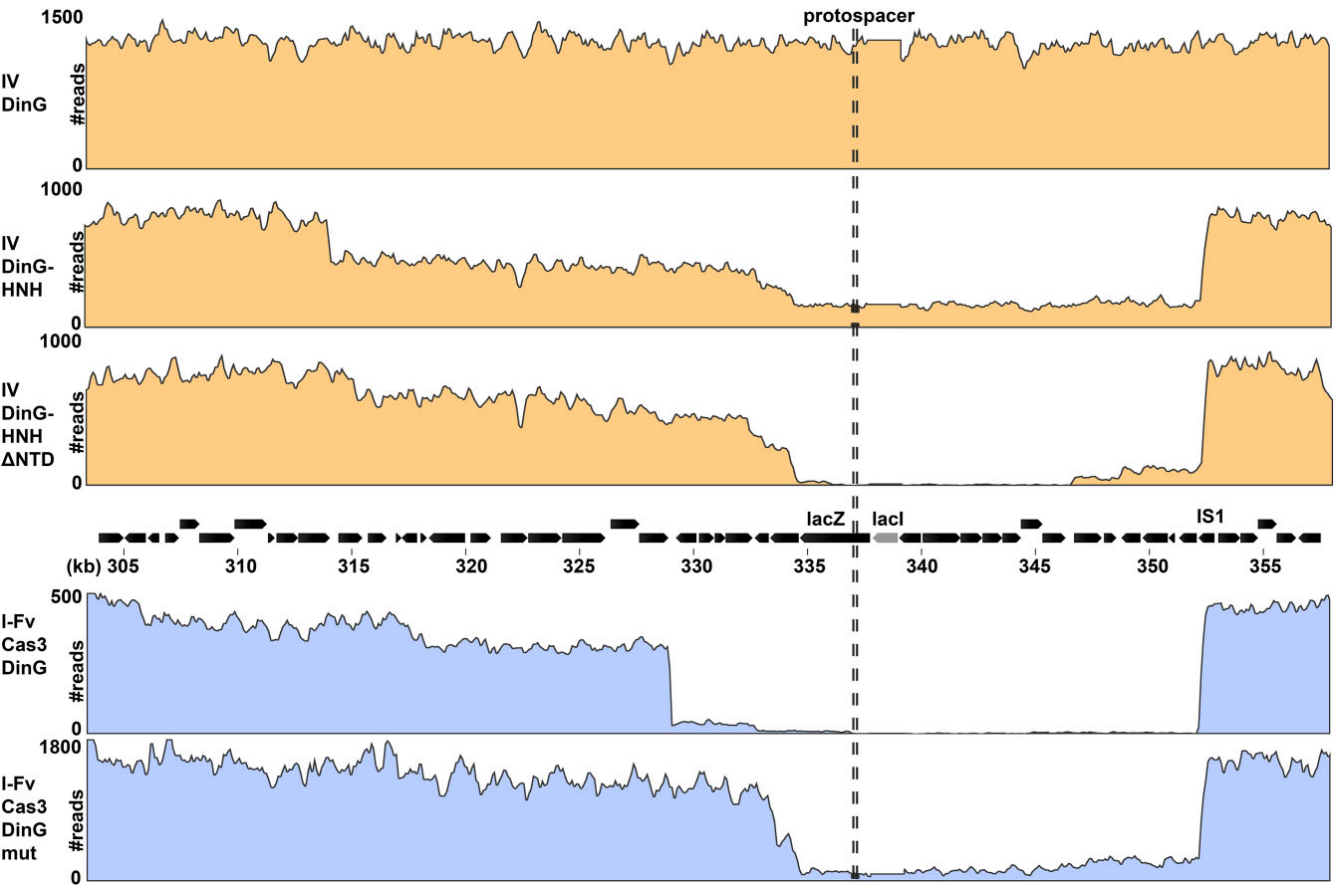
WGS analyses to identify deletions generated with engineered type IV-A1 and type I-Fv systems. Top: Coverage plots of the neighboring region of the *lacZ* protospacer indicating the length of deletions generated with the engineered type IV-A1 systems. The type IV-A1 system with CasDinG–HNH generated a deletion of ~43.8 kb and the type IV-A1 system with CasDinGΔNTD deletions ranging from 17.6–34.1 kb. Middle: genomic organization shows the genomic position with the *lacZ* protospacer (dashed line). Coverage plots of the neighboring region of the protospacer showing the deleted region indicated by the absence of reads, generated with type I-Fv systems. Type I-Fv system with CasDinG generated deletions ranging from 13.1 to 46.4 kb and with the mutant CasDinGK82A ranging from 17.5 to 19.3 kb. Coverage Plots were visualized with QIAGEN CLC Genomics Workbench, reads covering lacI have been excluded due to its additional presence in the employed expression vectors.

Having established that both systems could target and delete *lacZ*, we next investigated the extent of genome deletions using WGS, which also allows to distinguish between genomic deletions and repression of *lacZ* transcription. White colonies from the *lacZ* assays were pooled, and their genomic DNA was sequenced and mapped to an *E. coli* BL21-AI reference (Supplementary Fig. S2 and Fig. 4). In cells expressing the engineered type IV-A1 system with CasDinG–HNH, a large deletion of ~44 kb was observed (Supplementary Fig. S3 and  Supplementary Table S1). Interestingly, this was the only deletion detected, suggesting that the pooled colonies may have originated from a single edited cell, possibly due to early selection against CasDinG–HNH toxicity. Therefore, we repeated the experiment with individual transformants and observed a broader range of genomic deletions (Fig. 4 and Supplementary Table S1) spanning 18–47 kb. Some cells retained intact genomes but escaped CasDinG–HNH toxicity through deletion of the crRNA spacer or mutations in *dinG*. A control WGS experiment using the type IV-A1 effector with wild-type CasDinG (lacking the HNH fusion) did not produce genomic deletions (Fig. 4). The CasDinG–HNHΔNTD variant resulted in heterogeneous deletion sizes ranging from ~17 kb to over 44 kb. Some reads also matched undeleted wild-type DNA, suggesting incomplete or variable editing efficiency. These deletions consistently extended downstream of the *lacZ* target site into a nonessential genomic region. For the type I-Fv system, we observed deletion sizes up to 46.4 kb when CasDinG was present and up to 19.3 kb when the K82A mutant (Walker A motif lysine) was used (Fig. 4 and Supplementary Table S1). These results suggest that CasDinG enhances the processivity of the type I-Fv system and may contribute to bidirectional degradation or increased deletion span.

### Repair of CRISPR–Cas-induced DNA lesions

WGS revealed that deletions induced by both the engineered type IV-A1 and type I-Fv systems consistently terminated at a conserved site upstream of *lacZ* (Fig. 4). Sequence analysis of these junctions uncovered the presence of an inverted repeat (IRR) corresponding to the right end of an IS1A insertion sequence. This strongly suggests that IS1 elements act as genomic landmarks that halt the processive DNA degradation triggered by CasDinG–HNH or Cas3, potentially facilitating repair.

**Figure 5.**
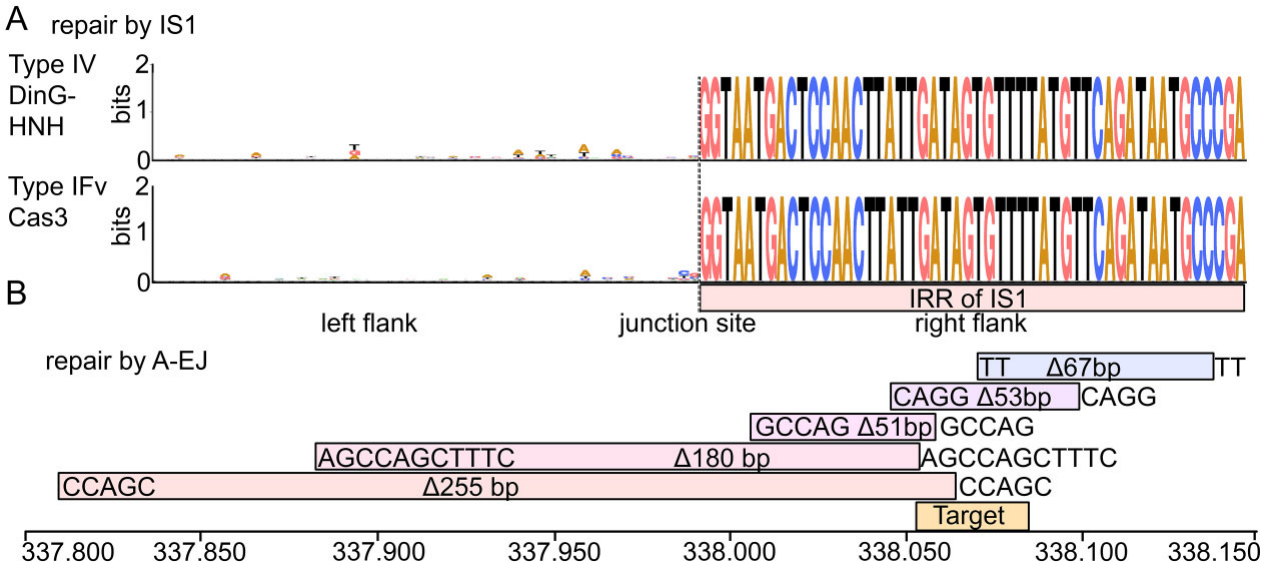
DNA repair mechanisms of CRISPR–Cas induced lesions in *E. coli*. (**A**) Sequence logo of flanking regions of all identified deletions generated with type IV-A1 CasDinG–HNH (top) or type I-Fv (bottom). A conserved IRR sequence of an IS1 sequence was identified at one flanking region for all deletions indicating its involvement in DNA repair. (**B**) Type IV-A1 generated deletions showed occasionally smaller deletions ranging from 51–255 bp. We identified short homologous sequence similarities between flanking regions and parts of the deleted sequence indicating a repair of the lesion by alternative end joining (A-EJ).

To further test this, we performed additional deletion assays targeting *dmpG*, a gene located between *lacZ* and the IS1A sequence, using the type I-Fv system. Colony PCRs were used to detect the presence or absence of adjacent genes and to localize the deletion boundaries. Regardless of whether the sense or antisense strand was targeted, deletions again terminated at the IS1A IRR, reinforcing the idea that this sequence element functions as a boundary for CRISPR–Cas-induced deletions (Supplementary Fig. S4). This suggests a model in which the IRR of IS1A may be involved in directing the repair of the resulting genomic lesion (Fig. 5A). The consistent halting of degradation at this site across different systems and targets points to a generalizable mechanism of repair influenced by MGEs.

In addition to large deletions, we occasionally observed smaller deletions (51–255 bp) among the *lacZ*-targeted white colonies in type IV-A1 CasDinG–HNH assays. Agarose gel electrophoresis revealed shortened PCR products, and sequencing of these products showed short stretches of 2–11 bp of microhomology at the deletion junctions (Fig. 5B). These features are characteristic of A-EJ, a DNA repair pathway that relies on microhomologous sequences to re-ligate broken DNA ends. A different target for the type IV-A–HNH system yielded both microhomologies and IS1 flanks (Supplementary Table S1) highlighting the universality of these repair solutions.

## Discussion

In this study, we present two class 1 CRISPR–Cas systems adapted for heterologous genome engineering: the minimal type I-Fv system from *S. putrefaciens* and an engineered type IV-A1 system based on the type IV-A1 system of *P. oleovorans*. Both systems employ compact effector complexes that target DNA and recruit helicase–nuclease proteins—Cas3 or CasDinG–HNH—for processive degradation. While previous work focused largely on type I-C systems (in *E. coli, Pseudomonas syringae, Klebsiella pneumoniae, Streptomyces coelicolor*, and *Bifidobacterium breve* [9, 46, 47]), our results expand the Class 1 genome editing toolkit with additional systems offering distinct properties and PAM specificities.

The type I-Fv system, which uses a simplified Cascade complex and a Cas2/3 fusion, was shown here to induce deletions of up to 46.4 kb in *E. coli*. This system targets a 5′-GG-3′ PAM and provides an alternative to the type I-C systems that identify a 5′-TTC-3′ PAM and require four Cas proteins (*cas3, cas5, cas7, cas8*). We also engineered a type IV-A1 CRISPR–Cas system by fusing an HNH nuclease domain to CasDinG, transforming it from a gene silencing effector into a DNA-cleaving enzyme. This engineered CasDinG–HNH system induced targeted deletions up to 43.8 kb and operates with a distinct 5′-AAG-3′ PAM, broadening the targeting scope available for class-1-based genome engineering. Both systems are plasmid-encoded, contain all required *cas* genes, and support easy spacer exchange via Golden Gate cloning [39].

Our findings also offer new perspectives on the evolution of CRISPR-associated DinG proteins. In the type I-Fv system, CasDinG was found to be dispensable, as genome deletions still occurred when it is mutated—albeit smaller. This suggests that CasDinG’s helicase activity may support DNA unwinding during the deletion process. In contrast, CasDinG is essential in type IV systems for CRISPRi activity unless the target lies directly in a promoter region [22, 16]. Here, CasDinG likely unwinds DNA near the target site, disrupting the promoter duplex and impairing RNA polymerase binding. The engineered CasDinG–HNH fusion demonstrates both the modularity of this system and provides a means to track CasDinG helicase activity via the HNH-mediated deletions. One such deletion spanned over 47 kb, consistent with the effective CRISPRi range observed for the native system [16]. Interestingly, the loss of the NTD of CasDinG was tolerated but resulted in smaller deletions, suggesting that this version could be useful for more precise engineering. Notably, CasDinG exhibits 5′→3′ helicase activity, opposite to the 3′→5′ directionality of Cas3. However, these mechanistic differences between type IV and type I CRISPRi complexes were not reflected in our genome deletion patterns, which showed bidirectional deletions and suggest that successful DNA repair serves as the predominant selection mechanism.

Our results also revealed that full-length CasDinG–HNH was more toxic than the version without the HNH fusion as bacterial growth was often impaired in the presence of this protein. Native CasDinG variants without nuclease activities could be an evolutionary consequence of the loss of toxic off-target DNase activities while retaining plasmid defense [12] and transcriptional repression functions [15]. In our experiments, bacterial survival correlated with loss of the CRISPR targeting sequence, indicating that early selection for an initial deletion may have limited the diversity of observed deletion outcomes.

Sequencing of the genome junctions created by both genome deletion systems revealed how *E. coli* repairs CRISPR-induced DNA damage. In type IV-A1-mediated deletions, we observed occasional small deletions of 51–255 bp flanked by 2–11 bp of microhomology (Fig. 5). Microhomologies were also noted earlier for deletions generated by the type I-C system in *P. aeruginosa* [9]. This pattern suggests repair via A-EJ, a RecBCD-dependent mechanism that uses limited homology and is often associated with LigA-mediated re-ligation [48]. In this context, CasDinG–HNH or Cas3 likely generates single-stranded overhangs that expose microhomologous regions to enable repair. The most frequently observed junctions involved an 11-nt microhomology stretch, supporting the idea that extended homology regions facilitate repair.

Beyond microhomology-mediated repair, we consistently detected that both genome engineering systems created deletions ending precisely at a conserved sequence which was revealed to be the inverted repeat IRR of the insertion sequence IS1. IS1 is a 768 bp element flanked by ~30 bp terminal inverted repeats (IRL and IRR) and encodes a transposase. The *E. coli* BL21 AI genome contains 28 IS1 elements, 17 of which have nearby direct repeats. We propose that CasDinG–HNH-induced deletions may eliminate direct repeats, creating fusions between genome fragments and the IRR sequence without obvious homology (Fig. 5). IS1 elements facilitate deletion repair either via (i) RecA-dependent homologous recombination between IS1 copies or (ii) RecA-independent abortive transposition [49].

Our results suggest that these IS1 elements serve as genetic anchors, limiting degradation and promoting repair through site-specific mechanisms—an effect observed previously near MGEs [50]. These observations have practical implications for genome engineering: IS elements could act as predictable stop points during Cas3- or CasDinG-mediated degradation, helping guide the design of controlled deletions. This could be especially useful in synthetic biology applications where genome minimization or streamlining is desired.

In conclusion, our study (i) expands the Class I CRISPR–Cas toolkit for genome engineering by introducing two new systems with distinct PAM specificities and functional properties, (ii) provides insights into the evolution of modular CasDinG proteins, and (iii) revealed how *E. coli* repairs class I CRISPR–Cas-induced DNA damage.

## Supplementary Material

gkaf1399_Supplemental_Files

## Data Availability

All data are available in the manuscript or the Supplementary data file. Raw data from WGS are available at the European Nucleotide Archive under the accession code PRJEB89807. All shell and R scripts generated for WGS analyses of genomic deletions are publicly available at https://github.com/VicenteBR/Deletion-boundary-detection--Klein-et-al--2025- and https://doi.org/10.5281/zenodo.17630329.

## Notes

Present address: Samuel Novak, Max Planck School Matter to Life, 69120 Heidelberg, Jahnstraße 29,Germany

Present Address: Lukas Pekarek, Excellence Cluster Physics of Life, Technical University Dresden, Arnoldstraße 18, 01307 Dresden, Germany
