# Supplementary material for: Type I-Fv and engineered type IV-A1 CRISPR–Cas effectors facilitate genome reduction in Escherichia coli
Source: Nucleic Acids Res. 2025 Dec 18;53(22):gkaf1399. doi: 10.1093/nar/gkaf1399 (PMC12714560; doi:10.1093/nar/gkaf1399)
Supplement: gkaf1399_Supplemental_Files [file gkaf1399_supplemental_files.zip › Klein NAR Supplementary information Nov25.pdf]

Supplementary Data

Type I-Fv and engineered Type IV-A CRISPR-Cas effectors facilitate genome reduction in *Escherichia coli*

Nathalie Klein[1], Mariana Sanchez-Londono[1], Meral M. Kara[1], José Vicente Gomes-Filho[1], Samuel Novak[2], Karim H. Kholeif[1], Lukas Pekarek[2], Neva Caliskan[2,4] and Lennart Randau*[1,3]

[1] Department of Biology, Philipps-Universität Marburg, Hans-Meerwein-Str. 6, 35043 Marburg, Germany
[2] Helmholtz Institute for RNA-based Infection Research (HIRI-HZI), Josef Schneider Str. 2, 97080, Würzburg, Germany
[3] Center for Synthetic Microbiology (SYNMIKRO), Karl-von-Frisch-Straße 14, 35043 Marburg, Germany
[4] Department of Biochemistry III, University of Regensburg, Universitätsstr. 31, 93053, Regensburg, Germany

* To whom correspondence should be addressed. Tel: +49 (0)6421 28 23320; Fax: +49 (0)6421-178 599; Email: lennart.randau@staff.uni-marburg.de

Present Address: Lukas Pekarek, Excellence Cluster Physics of Life, Technical University Dresden, Arnoldstraße 18, 01307 Dresden, Germany
Samuel Novak, Heidelberg University, 69117, Heidelberg, Germany

Supplementary data content

Supplementary Table S1: Genomic positions of deletions in *E. coli* generated with type I-Fv and engineered type IV-A systems

Supplementary Table S2: Plasmids used in this study

Supplementary Table S3: Oligonucleotides used for primer walking

Supplementary Table S4: Protospacer sequences of genomic targets in the *E. coli* genome

Supplementary Table S5: WGS data analysis

Supplementary Figure S1: C-trap measurement of labeled CasDinG in the absence of Type IV crRNPs

Supplementary Figure S2: Workflow for genomic targeting assays with engineered type IV-A1 and type I-Fv CRISPR-Cas systems

Supplementary Figure S3: Deletions of *E. coli* genomes with *lacZ* target (1st batch)

Supplementary Figure S4: Deletions of *E. coli* genomes with *dmpG* target

Supplementary Video S1: Visualization of CasDinG recruitment

Supplementary Table S1: Genomic positions of deletions in *E. coli* generated with type I-Fv and engineered type IV-A systems. Microhomologies are indicated in red. IRR and IRL sequences are indicated in blue. Counts indicate the numbers of times this specific junction was detected in WGS data.

	start	end	Deletion size	Counts	flank upstream (50 nt)	flank downstream (50 nt)
**IV-A CasDinG-HNH**	308820	352688	43867	Batch 1	ATATTGGCTCCAGTTCCGGCTGCGACATGCTGTATGGCATGGCCGATGGT	GGTAATGACTCCAACTTATTGATAGTGTTTTATGTTCAGATAATGCCCG
	314801	352688	37887	419	CGATTTTAGTCTTTTACAGATAGCAAATATCACACTTACAGGCGCTCGCC	GGTAATGACTCCAACTTATTGATAGTGTTTTATGTTCAGATAATGCCCG
	334149	352688	18539	70	AAACAGACCAGATAAATCGTCGCTGAAAAACGCACTTCAAACTGGCTGGT	GGTAATGACTCCAACTTATTGATAGTGTTTTATGTTCAGATAATGCCCG
	334966	352688	17722	63	GGTAATAATCCACAGCAGGTATTTGCGCAGCCCGAGTTTGTCAGAAAGCA	GGTAATGACTCCAACTTATTGATAGTGTTTTATGTTCAGATAATGCCCG
	317825	352688	34863	62	CGCGAATACACCAATAATTGCACCAATAACCCGACTGGCAACGCGTTGCA	GGTAATGACTCCAACTTATTGATAGTGTTTTATGTTCAGATAATGCCCG
	333528	352688	19160	62	CAATTCATGGTGTACAGGGTGTCCCGTAACGGAAAGAGTAACGTTGGGTG	GGTAATGACTCCAACTTATTGATAGTGTTTTATGTTCAGATAATGCCCG
	305481	352688	47207	60	TGATCGGTAAAGGCGTTCACTTTTTGCAGGGCTATTTGTACTCTCCGCCA	GGTAATGACTCCAACTTATTGATAGTGTTTTATGTTCAGATAATGCCCG
	317826	352688	34862	54	CGCGAATACACCAATAATTGCACCAATAACCCGACTGGCAACGCGTTGCA	GGTAATGACTCCAACTTATTGATAGTGTTTTATGTTCAGATAATGCCCG
	333437	352688	19251	48	TGGTGACGCCTGGATTAATAACCACATGACTTCCGATCCAGACGTTATTG	GGTAATGACTCCAACTTATTGATAGTGTTTTATGTTCAGATAATGCCCG
	334623	352688	18065	45	GTGGCAGAAGAGGGCGCATCCGTTTTGGCGAAAAAGAGTAAAACGGCGAG	GGTAATGACTCCAACTTATTGATAGTGTTTTATGTTCAGATAATGCCCG
	333367	352688	19321	39	GCCGCCACGACGTTTGGTGGAATGTCTTTTGTGACGATACTACCCGCGCC	GGTAATGACTCCAACTTATTGATAGTGTTTTATGTTCAGATAATGCCCG
	334890	352688	17798	34	ATCCTACTAAAATGTTGTATTGTAACAGTGGCCCGAAGATAAAAATAAAC	GGTAATGACTCCAACTTATTGATAGTGTTTTATGTTCAGATAATGCCCG
	308446	352688	44242	29	TCCGTTTTATCAGGCGTTAAATAAAGAACTACATATTAAACGACGGGCAG	GGTAATGACTCCAACTTATTGATAGTGTTTTATGTTCAGATAATGCCCG
	333947	352688	18741	29	ATTGATTGCTTAAGCGACTTCATTCACCTGACGACGCAGTAGAGAAAGCG	GGTAATGACTCCAACTTATTGATAGTGTTTTATGTTCAGATAATGCCCG
	334661	352688	18027	27	AAAACGGCGAGGATGAGTGCACAGCCAGAGCCCAGCCAGAAAACAAACTC	GGTAATGACTCCAACTTATTGATAGTGTTTTATGTTCAGATAATGCCCG
	333576	352688	19112	18	TGCAATCAGTACGTTATCACCGATTGTTACCGTGTAGTCATCGACAATGG	GGTAATGACTCCAACTTATTGATAGTGTTTTATGTTCAGATAATGCCCG
	333754	352688	18934	14	TTTTCAACTTCTGATGGATGCGAGTGATTAAACTCATACATTAACGTTTT	GGTAATGACTCCAACTTATTGATAGTGTTTTATGTTCAGATAATGCCCG
	334891	352688	17797	14	ATCCTACTAAAATGTTGTATTGTAACAGTGGCCCGAAGATAAAAATAAAG	GGTAATGACTCCAACTTATTGATAGTGTTTTATGTTCAGATAATGCCCG
	326790	352688	25898	11	TCAGGCCTACCCACTGTTTTTACACCGATAATTTTTCCCCCACCTTTTTG	GGTAATGACTCCAACTTATTGATAGTGTTTTATGTTCAGATAATGCCCG
	335159	352688	17529	10	AAAAGAATAAACCGAACATCCAAAAGTTTGTGTTTTTTAAATAGTACATA	GGTAATGACTCCAACTTATTGATAGTGTTTTATGTTCAGATAATGCCCG
	334953	352688	17735	9	ATCACTAACATGCCGGTAATAATCCACAGCAGGTATTTGCGCAGCCCGAG	GGTAATGACTCCAACTTATTGATAGTGTTTTATGTTCAGATAATGCCCG
	327613	352688	25075	8	CCAAACTGGCGGTGCTGGTGGCGATGGTGGCCTTTTTCCTCGGCAACTCG	GGTAATGACTCCAACTTATTGATAGTGTTTTATGTTCAGATAATGCCCG
	334622	352688	18066	8	CGTGGCAGAAGAGGGCGCATCCGTTTTGGCGAAAAAGAGTAAAACGGCGA	GGTAATGACTCCAACTTATTGATAGTGTTTTATGTTCAGATAATGCCCG
	327613	352688	25075	6	CCAAACTGGCGGTGCTGGTGGCGATGGTGGCCTTTTTCCTCGGCAACTCG	GGTAATGACTCCAACTTATTGATAGTGTTTTATGTTCAGATAATGCCCG
	333268	352688	19420	5	ATTTTTATAATTTAAACTGACGATTCAACTTTATAATCTTTGAAATAATA	GGTAATGACTCCAACTTATTGATAGTGTTTTATGTTCAGATAATGCCCG

**IV-A**	244367	245388	1021	36	ACGCAGCGAATTGAGCGGCATAACCTGAATCTGAGGCAGCACCTGGCACG	GCTGGGAAGATATAGAGGATTTTGGGGAAACACATCTCGATTTTTTAAAA
**CasDinG-**	244473	245319	846	30	TCGGGCATTATCTGAACATAAAACACTATCAATAAGTTGGAGTCATTACC	GGAAAGTGGAACATAAATTGTCAGATATTCTACTGTTGACTATTTGTGCC
**HNH**	244897	245149	252	104	GAGGAGGTTATTAATAGCAGGAAAAGCATCACGTGATGCTGATTTTTTGC	ACTAAATAATTCGCATTTTATGTTTAAAAATTGAGATATTCCTCATTACC
**2nd target**						
**tRNA-Asp**						
**IV-A CasDinG-HNH ΔNTD**	318492	352688	34195	Batch 1	AGATCACAAAGTGTCTTATTTCCGGTTACTGGCGTTTATGCCCTGACTGA	GGTAATGACTCCAACTTATTGATAGTGTTTTATGTTCAGATAATGCCCGA
	326812	352688	25875	Batch 1	ACACCGATAATTTTTCCCCCACCTTTTTGCACTCATTCATATAAAAAATA	GGTAATGACTCCAACTTATTGATAGTGTTTTATGTTCAGATAATGCCCGA
	333198	352688	19489	Batch 1	TTGTTCATGCCGGATGCGGCTAATGTAGATCGCTGAACTTGTAGGCCTGA	GGTAATGACTCCAACTTATTGATAGTGTTTTATGTTCAGATAATGCCCGA
	333553	352688	19134	Batch 1	CGTAACGGAAAGAGTAACGTTGGGTGCAATCAGTACGTTATCACCGATTG	GGTAATGACTCCAACTTATTGATAGTGTTTTATGTTCAGATAATGCCCGA
	333572	352688	19115	Batch 1	TTGGGTGCAATCAGTACGTTATCACCGATTGTTACCGTGTAGTCATCGAC	GGTAATGACTCCAACTTATTGATAGTGTTTTATGTTCAGATAATGCCCGA
	334876	352688	17811	Batch 1	AAATACCACCAACAATCGATCCTACTAAAATGTTGTATTGTAACAGTGGC	GGTAATGACTCCAACTTATTGATAGTGTTTTATGTTCAGATAATGCCCGA
	335001	352688	17686	Batch 1	GAGTTTGTCAGAAAGCAGACCAAACAGCGGTTGGAATAATAGCGAGAACA	GGTAATGACTCCAACTTATTGATAGTGTTTTATGTTCAGATAATGCCCGA
	335088	352688	17599	Batch 1	GCTGATATGGTTGATGTCATGTAGCCAAATCGGGAAAAACGGGAAGTAGG	GGTAATGACTCCAACTTATTGATAGTGTTTTATGTTCAGATAATGCCCGA
	334957	352688	17731	112	CTAACATGCCGGTAATAATCCACAGCAGGTATTTGCGCAGCCCGAGTTTG	GGTAATGACTCCAACTTATTGATAGTGTTTTATGTTCAGATAATGCCCGA
	315937	352688	36751	85	GCATCGTGGCTGATATAGAGATGATTCGGGCCGATCAAATTAATGAAGCC	GGTAATGACTCCAACTTATTGATAGTGTTTTATGTTCAGATAATGCCCGA
	333697	352688	18991	79	TCTACCCAGGCGTTTTCCCCTACCGTGGCAAACATTTCTTTAATCAGGCT	GGTAATGACTCCAACTTATTGATAGTGTTTTATGTTCAGATAATGCCCGA
	325593	352688	27095	58	GCGGATATGCGGTGGCGCTGGCGACCTCGATGGACACCATTTTTGGCGGC	GGTAATGACTCCAACTTATTGATAGTGTTTTATGTTCAGATAATGCCCGA
	334062	352688	18626	55	GATAAGCGCCCTGGAAACCGATGCTTTCATACATATTGCCCGCCAGTACA	GGTAATGACTCCAACTTATTGATAGTGTTTTATGTTCAGATAATGCCCGA
	325592	352688	27096	54	GCGGATATGCGGTGGCGCTGGCGACCTCGATGGACACCATTTTTGGCGGC	GGTAATGACTCCAACTTATTGATAGTGTTTTATGTTCAGATAATGCCCGA
	320152	352688	32536	46	TCTTCGGTAATGTAGCTACGTTGATCGAGGCGCAAATTAAAGGTTTTTTG	GGTAATGACTCCAACTTATTGATAGTGTTTTATGTTCAGATAATGCCCGA
	334796	352688	17892	43	GAAATTACTGCGACGGCTGACTTTCTCAATAAATGCCTCTACTGCTGGCG	GGTAATGACTCCAACTTATTGATAGTGTTTTATGTTCAGATAATGCCCGA
	333138	352688	19550	42	CCAGTACGTTTTCCGCAGCTGTGGGTCAAAGAGGCATGATGCGACGCTTG	GGTAATGACTCCAACTTATTGATAGTGTTTTATGTTCAGATAATGCCCGA
	334997	352688	17691	41	CCGAGTTTGTCAGAAAGCAGACCAAACAGCGGTTGGAATAATAGCGAGAA	GGTAATGACTCCAACTTATTGATAGTGTTTTATGTTCAGATAATGCCCGA
	308688	352688	44000	39	CCACATTATTAAGTGCCATAACGCTGGAAGCCGCATTACAACAACGCTGC	GGTAATGACTCCAACTTATTGATAGTGTTTTATGTTCAGATAATGCCCGA
	334764	352688	17924	39	ACACAGCCAAACATCCGCGCGCGACCAAATTCGAAATTACTGCGACGGCT	GGTAATGACTCCAACTTATTGATAGTGTTTTATGTTCAGATAATGCCCGA
	333244	352688	19444	37	CTGATAAGCGCAGCGTATCAGGCAATTTTTATAATTTAAACTGACGATTC	GGTAATGACTCCAACTTATTGATAGTGTTTTATGTTCAGATAATGCCCGA
	316946	352688	35742	36	TCTACCCTGTTTTTCCTGAATCACTGGTGATGTTATTGCTCTCTGTTCTG	GGTAATGACTCCAACTTATTGATAGTGTTTTATGTTCAGATAATGCCCGA
	334796	352688	17892	35	GAAATTACTGCGACGGCTGACTTTCTCAATAAATGCCTCTACTGCTGGCG	GGTAATGACTCCAACTTATTGATAGTGTTTTATGTTCAGATAATGCCCGA
	334889	352688	17799	34	GATCCTACTAAAATGTTGTATTGTAACAGTGGCCCGAAGATAAAAATAAA	GGTAATGACTCCAACTTATTGATAGTGTTTTATGTTCAGATAATGCCCGA
	335054	352688	17634	34	AATAGCGGCAAAAATAATACCCGTATCACTTTTGCTGATATGGTTGATGT	GGTAATGACTCCAACTTATTGATAGTGTTTTATGTTCAGATAATGCCCGA
	335041	352688	17647	33	GCGAGAACAGAGAAATAGCGGCAAAAATAATACCCGTATCACTTTTGCTG	GGTAATGACTCCAACTTATTGATAGTGTTTTATGTTCAGATAATGCCCGA
	334875	352688	17813	32	ATACCACCAACAATCGATCCTACTAAAATGTTGTATTGTAACAGTGGCCC	GGTAATGACTCCAACTTATTGATAGTGTTTTATGTTCAGATAATGCCCGA
	305289	352688	47399	28	ACGGGTTATGCGACCTATCGTTACTTGCAGGCGTTCCCGGTCGATTTTAT	GGTAATGACTCCAACTTATTGATAGTGTTTTATGTTCAGATAATGCCCGA
	333552	352688	19136	28	CGTAACGGAAAGAGTAACGTTGGGTGCAATCAGTACGTTATCACCGATTG	GGTAATGACTCCAACTTATTGATAGTGTTTTATGTTCAGATAATGCCCGA
	318397	352688	34291	25	AGGTAAACGGCGTGCAAAGGATCCATAGTGATTTCATCCATAAATAAGTG	GGTAATGACTCCAACTTATTGATAGTGTTTTATGTTCAGATAATGCCCGA
	330762	352688	21926	25	AGACTTTTACCTTATGACAATCGGCGAGTAGTCTGCCTCTCATTCCAGAG	GGTAATGACTCCAACTTATTGATAGTGTTTTATGTTCAGATAATGCCCGA

	333570	352688	19118	24	TTGGGTGCAATCAGTACGTTATCACCGATTGTTACCGTGTAGTCATCGAC	GGTAATGACTCCAACTTATTGATAGTGTTTTATGTTCAGATAATGCCCGA
	316946	352688	35742	23	TCTACCCTGTTTTTCCTGAATCACTGGTGATGTTATTGCTCTCTGTTCTG	GGTAATGACTCCAACTTATTGATAGTGTTTTATGTTCAGATAATGCCCGA
	334858	352688	17830	23	AACAAAAGCCTAGATAAATACCACCAACAATCGATCCTACTAAAATGTTG	GGTAATGACTCCAACTTATTGATAGTGTTTTATGTTCAGATAATGCCCGA
	314758	352688	37930	22	GTGTAGATAACTATTTGTCTACGTGAAAAGATCATCAGTTTTGCCGATTT	GGTAATGACTCCAACTTATTGATAGTGTTTTATGTTCAGATAATGCCCGA
	318481	352688	34207	22	GGGTGCGAGAGAGATCACAAAGTGTCTTATTTCCGGTTACTGGCGTTTAT	GGTAATGACTCCAACTTATTGATAGTGTTTTATGTTCAGATAATGCCCGA
	336545	352688	16143	22	CGGCGTATCGCCAAAATCACCGCCGTAAGCCGACCACGGGTTGCCGTTTT	GGTAATGACTCCAACTTATTGATAGTGTTTTATGTTCAGATAATGCCCGA
	308506	352688	44182	21	AGTAAACGAAAAACAAAATATCGCCGCAGCGGTCGCCAGTATGATGGGGT	GGTAATGACTCCAACTTATTGATAGTGTTTTATGTTCAGATAATGCCCGA
	334966	352688	17722	20	GGTAATAATCCACAGCAGGTATTTGCGCAGCCCGAGTTTGTCAGAAAGCA	GGTAATGACTCCAACTTATTGATAGTGTTTTATGTTCAGATAATGCCCGA
	333947	352688	18741	19	ATTGATTGCTTAAGCGACTTCATTCACCTGACGACGCAGTAGAGAAAGCG	GGTAATGACTCCAACTTATTGATAGTGTTTTATGTTCAGATAATGCCCGA
	334205	352688	18483	19	TTTAAAGCAGCCCACCAGCAGGAACGGTACTTCAAACATATGCAGCGTTT	GGTAATGACTCCAACTTATTGATAGTGTTTTATGTTCAGATAATGCCCGA
	316952	352688	35736	18	CTGTTTTTCCTGAATCACTGGTGATGTTATTGCTCTCTGTTCTGGACGTA	GGTAATGACTCCAACTTATTGATAGTGTTTTATGTTCAGATAATGCCCGA
	318591	352688	34097	18	AACTCTGGAGCCGTAATATGAAAATAATCTCTAAAATGTTAGTCGGTGCG	GGTAATGACTCCAACTTATTGATAGTGTTTTATGTTCAGATAATGCCCGA
	334781	352688	17907	18	CGCGCGACCAAATTCGAAATTACTGCGACGGCTGACTTTCTCAATAAATG	GGTAATGACTCCAACTTATTGATAGTGTTTTATGTTCAGATAATGCCCGA
	325058	352688	27630	17	AAACAGAAGAAGAGCGCACCTTTACCTTTCGTCAGCTGCATGACGAAGTG	GGTAATGACTCCAACTTATTGATAGTGTTTTATGTTCAGATAATGCCCGA
	332976	352688	19712	16	GGCAAGCTGGTGGCGTTTATGCAGGGAATCGGTTTTATCATCGCCGGGCT	GGTAATGACTCCAACTTATTGATAGTGTTTTATGTTCAGATAATGCCCGA
	333438	352688	19250	16	GTGACGCCTGGATTAATAACCACATGACTTCCGATCCAGACGTTATTGCC	GGTAATGACTCCAACTTATTGATAGTGTTTTATGTTCAGATAATGCCCGA
	334857	352688	17831	16	AAACAAAAGCCTAGATAAATACCACCAACAATCGATCCTACTAAAATGTT	GGTAATGACTCCAACTTATTGATAGTGTTTTATGTTCAGATAATGCCCGA
	323195	352688	29493	15	TGGGCGGCGCACATTATCGAACAACGTCAGGACAACAAAATTATCCGTCC	GGTAATGACTCCAACTTATTGATAGTGTTTTATGTTCAGATAATGCCCGA
	334961	352688	17727	15	ATGCCGGTAATAATCCACAGCAGGTATTTGCGCAGCCCGAGTTTGTCAGA	GGTAATGACTCCAACTTATTGATAGTGTTTTATGTTCAGATAATGCCCGA
	316950	352688	35738	14	CTGTTTTTCCTGAATCACTGGTGATGTTATTGCTCTCTGTTCTGGACGTA	GGTAATGACTCCAACTTATTGATAGTGTTTTATGTTCAGATAATGCCCGA
	317111	352688	35577	14	TTTAGTTCAGAAAGGGTTCCACCTGCAAATTGTTATTCCACCTGAGAGTG	GGTAATGACTCCAACTTATTGATAGTGTTTTATGTTCAGATAATGCCCGA
	317478	352688	35210	14	AGAGCAGACGACAACCAGGATTGTTGTATGGTACGGGGTTCGAGGCCTCG	GGTAATGACTCCAACTTATTGATAGTGTTTTATGTTCAGATAATGCCCGA
	318591	352688	34097	14	AACTCTGGAGCCGTAATATGAAAATAATCTCTAAAATGTTAGTCGGTGCG	GGTAATGACTCCAACTTATTGATAGTGTTTTATGTTCAGATAATGCCCGA
	333443	352688	19245	14	CGCCTGGATTAATAACCACATGACTTCCGATCCAGACGTTATTGCCAATC	GGTAATGACTCCAACTTATTGATAGTGTTTTATGTTCAGATAATGCCCGA
	335192	352688	17496	14	GTTTTTTAAATAGTACATAATGGATTTCCTTACGCGAAATACGGGCAGAC	GGTAATGACTCCAACTTATTGATAGTGTTTTATGTTCAGATAATGCCCGA
	323194	352688	29494	13	TGGGCGGCGCACATTATCGAACAACGTCAGGACAACAAAATTATCCGTCC	GGTAATGACTCCAACTTATTGATAGTGTTTTATGTTCAGATAATGCCCGA
	332545	352688	20143	13	GCGCCCGCGAGGTCGCCTCTTCCCACAAGACAACAACCACTCCGGTTCGC	GGTAATGACTCCAACTTATTGATAGTGTTTTATGTTCAGATAATGCCCGA
	334737	352688	17951	13	ACAATCGAGGCACACAGCGCCCAGCCAACACAGCCAAACATCCGCGCGCG	GGTAATGACTCCAACTTATTGATAGTGTTTTATGTTCAGATAATGCCCGA
	334807	352688	17881	13	ACGGCTGACTTTCTCAATAAATGCCTCTACTGCTGGCGCACCGGCGTTAA	GGTAATGACTCCAACTTATTGATAGTGTTTTATGTTCAGATAATGCCCGA
	317808	352688	34880	11	TTCGTAAATCAGGCGTAGCGCGAATACACCAATAATTGCACCAATAACCC	GGTAATGACTCCAACTTATTGATAGTGTTTTATGTTCAGATAATGCCCGA
	318707	352688	33981	11	GAATCGCAGTATGAAAAAATAGGTGATATTTCAACCAGTAATGAAATGTC	GGTAATGACTCCAACTTATTGATAGTGTTTTATGTTCAGATAATGCCCGA
	337126	352688	15562	11	CGCACAGCGTGTACCACAGCGGATGGTTCGGATAATGCGAACAGCGCACG	GGTAATGACTCCAACTTATTGATAGTGTTTTATGTTCAGATAATGCCCGA
	318705	352688	33983	9	GAATCGCAGTATGAAAAAATAGGTGATATTTCAACCAGTAATGAAATGTC	GGTAATGACTCCAACTTATTGATAGTGTTTTATGTTCAGATAATGCCCGA
	284360	352688	68328	8	GCTGTGTGATGGACTAGTAAAAAAACAAGTTTTAAGGGTAAGGGATAGAA	GGTAATGACTCCAACTTATTGATAGTGTTTTATGTTCAGATAATGCCCGA
	316742	352688	35946	6	AATTAGTGCAGTTTATCGATTCAGTGCGAACAGATTGCAAATGTATTCTT	GGTAATGACTCCAACTTATTGATAGTGTTTTATGTTCAGATAATGCCCGA
	317807	352688	34881	6	TTCGTAAATCAGGCGTAGCGCGAATACACCAATAATTGCACCAATAACCC	GGTAATGACTCCAACTTATTGATAGTGTTTTATGTTCAGATAATGCCCGA

**I-Fv DinG**	306217	352688	46470	Batch 1	AAGCGTGCGGTGTGTTTGGGCGTATAAAGCAGCTGATTAATACAAATGCG	GGTAATGACTCCAACTTATTGATAGTGTTTTATGTTCAGATAATGCCCGA
	329435	352688	23252	Batch 1	CAAACCACCGTATATCTGGAGCAGCCAGAAGCCATCGATTACAAACGTTG	GGTAATGACTCCAACTTATTGATAGTGTTTTATGTTCAGATAATGCCCGA
	333103	352688	19584	Batch 1	GTCGTTGGGCTGATGATCATAACCCTGCGTTTTGCACCAGTACGTTTTCC	GGTAATGACTCCAACTTATTGATAGTGTTTTATGTTCAGATAATGCCCGA
	333210	352688	19477	Batch 1	GGATGCGGCTAATGTAGATCGCTGAACTTGTAGGCCTGATAAGCGCAGCG	GGTAATGACTCCAACTTATTGATAGTGTTTTATGTTCAGATAATGCCCGA
	337357	352688	15330	Batch 1	TCTGCTTCAATCAGCGTGCCGTCGGCGGTGTGCAGTTCAACCACCGCACG	GGTAATGACTCCAACTTATTGATAGTGTTTTATGTTCAGATAATGCCCGA
	334796	352688	17892	26	GAAATTACTGCGACGGCTGACTTTCTCAATAAATGCCTCTACTGCTGGCG	GGTAATGACTCCAACTTATTGATAGTGTTTTATGTTCAGATAATGCCCGA
	334817	352688	17871	26	TTTCTCAATAAATGCCTCTACTGCTGGCGCACCGGCGTTAAAACAAAAGC	GGTAATGACTCCAACTTATTGATAGTGTTTTATGTTCAGATAATGCCCGA
	334844	352688	17844	25	CGCACCGGCGTTAAAACAAAAGCCTAGATAAATACCACCAACAATCGATC	GGTAATGACTCCAACTTATTGATAGTGTTTTATGTTCAGATAATGCCCGA
	334979	352688	17709	25	CAGCAGGTATTTGCGCAGCCCGAGTTTGTCAGAAAGCAGACCAAACAGCG	GGTAATGACTCCAACTTATTGATAGTGTTTTATGTTCAGATAATGCCCGA
	333570	352688	19118	24	GTTGGGTGCAATCAGTACGTTATCACCGATTGTTACCGTGTAGTCATCGA	GGTAATGACTCCAACTTATTGATAGTGTTTTATGTTCAGATAATGCCCGA
	327264	352688	25424	22	CAGGTTGGCTGGTTTGGCGTCGGTGTGGCGATGTTTGCCATTCCGGTGGG	GGTAATGACTCCAACTTATTGATAGTGTTTTATGTTCAGATAATGCCCGA
	314306	352688	38382	20	GGCGTAAAAGTGATGACTGGCACTGACAGCGTTATCGACCACTGGTCGCC	GGTAATGACTCCAACTTATTGATAGTGTTTTATGTTCAGATAATGCCCGA
	334936	352688	17752	20	TAAAGAACGGCGCAAACATCACTAACATGCCGGTAATAATCCACAGCAGG	GGTAATGACTCCAACTTATTGATAGTGTTTTATGTTCAGATAATGCCCGA
	327264	352688	25424	19	CAGGTTGGCTGGTTTGGCGTCGGTGTGGCGATGTTTGCCATTCCGGTGGG	GGTAATGACTCCAACTTATTGATAGTGTTTTATGTTCAGATAATGCCCGA
	333859	352688	18829	19	GCTTTTATTCTTTCTGTCATTGGCATGTTCATTGCGATCATTCCGTCCTG	GGTAATGACTCCAACTTATTGATAGTGTTTTATGTTCAGATAATGCCCGA
	333223	352688	19465	18	TAGATCGCTGAACTTGTAGGCCTGATAAGCGCAGCGTATCAGGCAATTTT	GGTAATGACTCCAACTTATTGATAGTGTTTTATGTTCAGATAATGCCCGA
	308896	352688	43792	17	GAATTATGTCAGTTAAAATAGTCATTAAACCGAATACCTATTTTGATTCT	GGTAATGACTCCAACTTATTGATAGTGTTTTATGTTCAGATAATGCCCGA
	334713	352688	17975	16	TTATTGATGGTGAACATGATGCCGACAATCGAGGCACACAGCGCCCAGCC	GGTAATGACTCCAACTTATTGATAGTGTTTTATGTTCAGATAATGCCCGA
	334956	352688	17732	16	ACTAACATGCCGGTAATAATCCACAGCAGGTATTTGCGCAGCCCGAGTTT	GGTAATGACTCCAACTTATTGATAGTGTTTTATGTTCAGATAATGCCCGA
	335148	352688	17540	16	AAAGTAAAAGAAAAAGAATAAACCGAACATCCAAAAGTTTGTGTTTTTTA	GGTAATGACTCCAACTTATTGATAGTGTTTTATGTTCAGATAATGCCCGA
	333472	352688	19216	13	ATCCAGACGTTATTGCCAATCGTTATCGGAAAAGAGTACATCTCGCCGTT	GGTAATGACTCCAACTTATTGATAGTGTTTTATGTTCAGATAATGCCCGA
	334686	352688	18002	13	CCAGAGCCCAGCCAGAAAACAAACTGATTATTGATGGTGAACATGATGCC	GGTAATGACTCCAACTTATTGATAGTGTTTTATGTTCAGATAATGCCCGA
	331861	352688	20827	11	AATTTGGCGATGGCATTATTAGCGCGATTAACTTCAAACTCGACGTTAAC	GGTAATGACTCCAACTTATTGATAGTGTTTTATGTTCAGATAATGCCCGA
	333646	352688	19042	11	AAATTGCGGCCTATATGGATGTTGGAACCGTAAGAGAAATAGACAGGCGG	GGTAATGACTCCAACTTATTGATAGTGTTTTATGTTCAGATAATGCCCGA
	334793	352688	17895	11	TTCGAAATTACTGCGACGGCTGACTTTCTCAATAAATGCCTCTACTGCTG	GGTAATGACTCCAACTTATTGATAGTGTTTTATGTTCAGATAATGCCCGA
	331526	352688	21162	10	AGTCACAAATTAACCGCAATATTCGTCTTGATCTTGCCGATGCCATTTTG	GGTAATGACTCCAACTTATTGATAGTGTTTTATGTTCAGATAATGCCCGA
	335254	352688	17434	10	GTTATTATTATTTTTGACACCAGACCAACTGGTAATGGTAGCGACCGGCG	GGTAATGACTCCAACTTATTGATAGTGTTTTATGTTCAGATAATGCCCGA
	317125	352688	35563	9	GGTTCCACCTGCAAATTGTTATTCCACCTGAGAGTGAATCGCAATGCAGG	GGTAATGACTCCAACTTATTGATAGTGTTTTATGTTCAGATAATGCCCGA
	327351	352688	25337	9	GTTTCCGGTTTACTGATGACCGTCACCGTCTTTTTTGGCATTTCGGCGCT	GGTAATGACTCCAACTTATTGATAGTGTTTTATGTTCAGATAATGCCCGA
	328829	352688	23859	9	GTCTCATCGACGTTCACTGTGATGAGATCGATGACGAGCAGTCGCGCTTT	GGTAATGACTCCAACTTATTGATAGTGTTTTATGTTCAGATAATGCCCGA
	334804	352688	17884	9	TGCGACGGCTGACTTTCTCAATAAATGCCTCTACTGCTGGCGCACCGGCG	GGTAATGACTCCAACTTATTGATAGTGTTTTATGTTCAGATAATGCCCGA
	317125	352688	35563	8	GGTTCCACCTGCAAATTGTTATTCCACCTGAGAGTGAATCGCAATGCAGG	GGTAATGACTCCAACTTATTGATAGTGTTTTATGTTCAGATAATGCCCGA
	333565	352688	19123	8	GTAACGTTGGGTGCAATCAGTACGTTATCACCGATTGTTACCGTGTAGTC	GGTAATGACTCCAACTTATTGATAGTGTTTTATGTTCAGATAATGCCCGA
	334107	352688	18581	8	AGTACAGACATAAAAATCATCGCCAGTTGCTTAAAGAAGCAGAAACAGAC	GGTAATGACTCCAACTTATTGATAGTGTTTTATGTTCAGATAATGCCCGA
	306077	352688	46611	7	CGCCGTCCAGTAGTGTATCGGGTGCGCCGATGGCGATATTGGCCTGATTA	GGTAATGACTCCAACTTATTGATAGTGTTTTATGTTCAGATAATGCCCGA
	306125	352688	46563	7	TATTAATAATCGCATCCCAGACGCCGTTATACACTTCGGTGGTGACGGTA	GGTAATGACTCCAACTTATTGATAGTGTTTTATGTTCAGATAATGCCCGA

	318604	352688	34084	7	TAATATGAAAATAATCTCTAAAATGTTAGTCGGTGCGTTAGCGTTAGCCG	GGTAATGACTCCAACTTATTGATAGTGTTTTATGTTCAGATAATGCCCGA
	318915	352688	33773	7	TAAGTTTATCGGTGATCATCTATTGAAATTTATGCCGGATAAAGTGTTCG	GGTAATGACTCCAACTTATTGATAGTGTTTTATGTTCAGATAATGCCCGA
	327351	352688	25337	7	GTTTCCGGTTTACTGATGACCGTCACCGTCTTTTTTGGCATTTCGGCGCT	GGTAATGACTCCAACTTATTGATAGTGTTTTATGTTCAGATAATGCCCGA
	333209	352688	19479	7	GGATGCGGCTAATGTAGATCGCTGAACTTGTAGGCCTGATAAGCGCAGCG	GGTAATGACTCCAACTTATTGATAGTGTTTTATGTTCAGATAATGCCCGA
	331861	352688	20827	6	AAATTTGGCGATGGCATTATTAGCGCGATTAACTTCAAACTCGACGTTAA	GGTAATGACTCCAACTTATTGATAGTGTTTTATGTTCAGATAATGCCCGA
	318339	352688	34349	5	ACCACAAAGAGATTGGCTCCCGGATTAAAAAAAGTAATCACGAACAGTCC	GGTAATGACTCCAACTTATTGATAGTGTTTTATGTTCAGATAATGCCCGA
	318340	352688	34348	5	ACCACAAAGAGATTGGCTCCCGGATTAAAAAAAGTAATCACGAACAGTCC	GGTAATGACTCCAACTTATTGATAGTGTTTTATGTTCAGATAATGCCCGA
	318604	352688	34084	5	TAATATGAAAATAATCTCTAAAATGTTAGTCGGTGCGTTAGCGTTAGCCG	GGTAATGACTCCAACTTATTGATAGTGTTTTATGTTCAGATAATGCCCGA
	333410	352688	19278	5	CCGCGCCAATAACAGAATTATCCCCGATGGTGACGCCTGGATTAATAACC	GGTAATGACTCCAACTTATTGATAGTGTTTTATGTTCAGATAATGCCCGA
	334841	352688	17847	5	TGGCGCACCGGCGTTAAAACAAAAGCCTAGATAAATACCACCAACAATCG	GGTAATGACTCCAACTTATTGATAGTGTTTTATGTTCAGATAATGCCCGA
**I-Fv CasDinG-K82A**	333307	352688	19380	Batch 1	GGTTTTATCATCGCCGGGCTTGCCCCGTGGTTTTCTGGCGTGCTGCGTAG	GGTAATGACTCCAACTTATTGATAGTGTTTTATGTTCAGATAATGCCCGA
	333354	352687	19332	Batch 1	ACAAGGAACGCCAGCCGCCACGACGTTTGGTGGAATGTCTTTTGTGACGA	GGTAATGACTCCAACTTATTGATAGTGTTTTATGTTCAGATAATGCCCGA
	334028	352688	18659	Batch 1	TTAAGGTGAAGCCCAGCGCCACCAGACCCAGCACCAGATAAGCGCCCTGG	GGTAATGACTCCAACTTATTGATAGTGTTTTATGTTCAGATAATGCCCGA
	334041	352688	18646	Batch 1	CAGCGCCACCAGACCCAGCACCAGATAAGCGCCCTGGAAACCGATGCTTT	GGTAATGACTCCAACTTATTGATAGTGTTTTATGTTCAGATAATGCCCGA
	334060	352688	18627	Batch 1	ACCAGATAAGCGCCCTGGAAACCGATGCTTTCATACATATTGCCCGCCAG	GGTAATGACTCCAACTTATTGATAGTGTTTTATGTTCAGATAATGCCCGA
	334714	352688	17973	Batch 1	TTATTGATGGTGAACATGATGCCGACAATCGAGGCACACAGCGCCCAGCC	GGTAATGACTCCAACTTATTGATAGTGTTTTATGTTCAGATAATGCCCGA
	334905	352688	17782	Batch 1	GTTGTATTGTAACAGTGGCCCGAAGATAAAAATAAAGAACGGCGCAAACA	GGTAATGACTCCAACTTATTGATAGTGTTTTATGTTCAGATAATGCCCGA
	334984	352688	17703	Batch 1	AGGTATTTGCGCAGCCCGAGTTTGTCAGAAAGCAGACCAAACAGCGGTTG	GGTAATGACTCCAACTTATTGATAGTGTTTTATGTTCAGATAATGCCCGA
	335095	352688	17592	Batch 1	TGGTTGATGTCATGTAGCCAAATCGGGAAAAACGGGAAGTAGGCTCCCAT	GGTAATGACTCCAACTTATTGATAGTGTTTTATGTTCAGATAATGCCCGA
	333591	352688	19097	51	ATCACCGATTGTTACCGTGTAGTCATCGACAATGGTTAAATTGAAATTTG	GGTAATGACTCCAACTTATTGATAGTGTTTTATGTTCAGATAATGCCCGA
	335172	352688	17516	36	CCGAACATCCAAAAGTTTGTGTTTTTTAAATAGTACATAATGGATTTCCT	GGTAATGACTCCAACTTATTGATAGTGTTTTATGTTCAGATAATGCCCGA
	333314	352688	19374	34	AATAGTGCTTATCCCGGTCGTTTATTTCGCGAATAACCCGACAAGGAACG	GGTAATGACTCCAACTTATTGATAGTGTTTTATGTTCAGATAATGCCCGA
	334121	352688	18567	31	AATCATCGCCAGTTGCTTAAAGAAGCAGAAACAGACCAGATAAATCGTCG	GGTAATGACTCCAACTTATTGATAGTGTTTTATGTTCAGATAATGCCCGA
	317167	352688	35521	30	AATGCAGGTAATCATTTCATCTGTATATCTGTATGCCAACAGGCATTCCA	GGTAATGACTCCAACTTATTGATAGTGTTTTATGTTCAGATAATGCCCGA
	333559	352688	19129	26	GAAAGAGTAACGTTGGGTGCAATCAGTACGTTATCACCGATTGTTACCGT	GGTAATGACTCCAACTTATTGATAGTGTTTTATGTTCAGATAATGCCCGA
	334957	352688	17731	26	CTAACATGCCGGTAATAATCCACAGCAGGTATTTGCGCAGCCCGAGTTTG	GGTAATGACTCCAACTTATTGATAGTGTTTTATGTTCAGATAATGCCCGA
	303462	352688	49226	20	AAACTTTCCTGTTAGAAGGCAGCCAGGGCAACGACCTGATGGACTACAGC	GGTAATGACTCCAACTTATTGATAGTGTTTTATGTTCAGATAATGCCCGA
	334817	352688	17871	20	TTTCTCAATAAATGCCTCTACTGCTGGCGCACCGGCGTTAAAACAAAAGC	GGTAATGACTCCAACTTATTGATAGTGTTTTATGTTCAGATAATGCCCGA
	333806	352688	18882	18	CACGAAGTCTTTTTTCCGGTAAGCCTTCGCACATATCGGTAAATAGCTTG	GGTAATGACTCCAACTTATTGATAGTGTTTTATGTTCAGATAATGCCCGA
	334722	352688	17966	18	GTGAACATGATGCCGACAATCGAGGCACACAGCGCCCAGCCAACACAGCC	GGTAATGACTCCAACTTATTGATAGTGTTTTATGTTCAGATAATGCCCGA
	334898	352688	17790	18	TAAAATGTTGTATTGTAACAGTGGCCCGAAGATAAAAATAAAGAACGGCG	GGTAATGACTCCAACTTATTGATAGTGTTTTATGTTCAGATAATGCCCGA
	334807	352688	17881	17	ACGGCTGACTTTCTCAATAAATGCCTCTACTGCTGGCGCACCGGCGTTAA	GGTAATGACTCCAACTTATTGATAGTGTTTTATGTTCAGATAATGCCCGA
	334771	352688	17917	16	AACATCCGCGCGCGACCAAATTCGAAATTACTGCGACGGCTGACTTTCTC	GGTAATGACTCCAACTTATTGATAGTGTTTTATGTTCAGATAATGCCCGA
	316988	352688	35700	15	CTGTTCTGGACGTAAAAACATACAGAGATGATGCGCGATTAAATGAATGG	GGTAATGACTCCAACTTATTGATAGTGTTTTATGTTCAGATAATGCCCGA
	335182	352688	17506	13	AAAAGTTTGTGTTTTTTAAATAGTACATAATGGATTTCCTTACGCGAAAT	GGTAATGACTCCAACTTATTGATAGTGTTTTATGTTCAGATAATGCCCGA

	316081	352688	36607	12	TTTAACATATTAACTTCGCTCTCCACTTAACTTTTTAGTTAAGGAGAGCG	GGTAATGACTCCAACTTATTGATAGTGTTTTATGTTCAGATAATGCCCGA
	318361	352688	34327	10	GGATTAAAAAAAGTAATCACGAACAGTCCTACGGTCAGGTAAACGGCGTG	GGTAATGACTCCAACTTATTGATAGTGTTTTATGTTCAGATAATGCCCGA
	333649	352688	19039	10	TTGCGGCCTATATGGATGTTGGAACCGTAAGAGAAATAGACAGGCGGTTC	GGTAATGACTCCAACTTATTGATAGTGTTTTATGTTCAGATAATGCCCGA
	304452	352688	48236	9	AATCACTCATACTGAACAGCGATAAAAGATAAAGGTGTGTTCATGAATTC	GGTAATGACTCCAACTTATTGATAGTGTTTTATGTTCAGATAATGCCCGA
	318361	352688	34327	9	GGATTAAAAAAAGTAATCACGAACAGTCCTACGGTCAGGTAAACGGCGTG	GGTAATGACTCCAACTTATTGATAGTGTTTTATGTTCAGATAATGCCCGA
	334249	352688	18439	9	GCGTTTTCAGAATAACCACTTCCAGCGCTGAGGTGGCGAACGATGAGCCA	GGTAATGACTCCAACTTATTGATAGTGTTTTATGTTCAGATAATGCCCGA
	333693	352688	18995	8	TTCTACCCAGGCGTTTTCCCCTACCGTGGCAAACATTTCTTTAATCAGGC	GGTAATGACTCCAACTTATTGATAGTGTTTTATGTTCAGATAATGCCCGA
	317413	352688	35275	7	CACTCAAGCCTGACAGAAAATATGCTGTAAGGCTCATATCAAAAACCGCC	GGTAATGACTCCAACTTATTGATAGTGTTTTATGTTCAGATAATGCCCGA
	316989	352688	35699	6	TGTTCTGGACGTAAAAACATACAGAGATGATGCGCGATTAAATGAATGGA	GGTAATGACTCCAACTTATTGATAGTGTTTTATGTTCAGATAATGCCCGA
	317414	352688	35274	5	ACTCAAGCCTGACAGAAAATATGCTGTAAGGCTCATATCAAAAACCGCCA	GGTAATGACTCCAACTTATTGATAGTGTTTTATGTTCAGATAATGCCCGA
	318838	352688	33850	5	GACAATAAGATCCACGGCACGGCAAATATTTATAAGAAGAAGTAATTCTC	GGTAATGACTCCAACTTATTGATAGTGTTTTATGTTCAGATAATGCCCGA
	330552	352688	22136	5	CGAATCGTTCGCCCGCTACGGTCAAACAGCGGCACGCCTAAACTCTCCTC	GGTAATGACTCCAACTTATTGATAGTGTTTTATGTTCAGATAATGCCCGA
	334689	352688	17999	5	GAGCCCAGCCAGAAAACAAACTGATTATTGATGGTGAACATGATGCCGAC	GGTAATGACTCCAACTTATTGATAGTGTTTTATGTTCAGATAATGCCCGA

Supplementary Table S2: Plasmids used in this study.

Plasmids	Description	Features	Reference
pAG19	pET-20b(+) derivative plasmid with C-terminally hexa-histidine tagged *casdinG*	Amp^R, T7 promoter, pBR322 origin	(Čepaitė et al. 2024)
pNK25	pRSF-Duet^{TM}1 derivative; MCS1: Type IV-A1 *cas* genes with C-terminally hexa-histidine tagged *cas8*; MCS2: *casdinG*		(Čepaitė et al. 2024)
pNK27	pRSF-Duet^{TM}1 derivative; MCS1: Type IV-A1 *cas* genes with C-terminally hexa-histidine tagged *cas8*		(Čepaitė et al. 2024)
pSR13	pETDuet™-1 derivative; MCS1: Type IV-A1 *cas* genes from *P.oleovorans* (*cas6*, *cas8*, *cas7*, *cas5*); MCS2: *casdinG*)	Amp^R, 2x T7 promoter, pBR322 origin, *lacI*	(Rust und Randau 2025)
pSR54	pCDF-Duet™-1 derivative; spacer targeting *lacZ* in *E. coli* BL21-AI;	Sm^R, 2x T7 promoter, CDF origin, *lacI*	(Guo et al. 2022)
pSR56	pCDF-Duet™-1 derivative; MCS1: Type IV-A1 repeat-spacer-repeat fragment with BseRI restriction site on the spacer. Used as non-targeting spacer	Sm^R, 2x T7 promoter, CDF origin, *lacI*	(Guo et al. 2022)
pHS1184_NZ_CP025815	pET45b (+) encoding *Sulfitobacter sp.* JL08 *casdinG* comprising HNH domain	Amp^R, T7 pro-moter, pBR322 origin, *lacI*	Addgene Plasmid#205965
pNK103	pSR13 derivative; MCS1: Type IV-A1 CRISPR-Cas from *P.oleovorans* (*cas6*, *cas8*, *cas7*, *cas5*); MCS2: *casdinGΔNTD*		This study
pNK104	pSR13 derivative; MCS1: Type IV-A1 CRISPR-Cas from *P.oleovorans* (*cas6*, *cas8*, *cas7*, *cas5*); MCS2: *casdinG*+HNH domain of *Sulfitobacter sp.* JL08		This study
pNK105	pSR13 derivative; MCS1: Type IV-A1 CRISPR-Cas from *P.oleovorans* (*cas6*, *cas8*, *cas7*, *cas5*);		This study

	MCS2: *casdinG*ΔNTD +HNH domain of *Sulfitobacter sp.* JL08		
pCas4	pRFSDuet™-1 derivative; *cas* (*cas3*, *cas7*, *cas5*, *cas6*) genes of Type I-Fv of *S. putrefaciens*	Km^R, 2x PT7, T7 terminator, RSF origin, *lacI*	(Gleditzsch et al. 2016)
pCDF-Duet™-1	Generation of pMK1.1	Sm^R, 2x T7 promoter, CDF origin, *lacI*	Novagen
pMK1.1	pCDF-Duet™-1 derivative; MCS1: type I-Fv repeat-spacer-repeat fragment with BseRI restriction site on the spacer		This study
pMK1	pMK1.1 derivative; MCS1: type I-Fv repeat-spacer-repeat fragment with BseRI restriction site on the spacer; MCS2: *casdinG*		This study
pMK3	pMK1 derivative; MCS1: repeat-spacer-repeat fragment with *lacZ* spacer; MCS2: *casdinG*		This study
pMK4	pMK1 derivative; MCS1: type I-Fv repeat-spacer-repeat fragment with BseRI restriction site on the spacer; MCS2: *casdinGK82A*		This study
pMK5	pMK3 derivative; MCS1: repeat-spacer-repeat fragment with *lacZ* spacer; MCS2: *casdinGK82A*		This study
pMK25	pMK1 derivative, MCS1: repeat-spacer-repeat fragment with *dmpG* spacer targeting coding strand; MCS2: *casdinG*		This study
pMK26	pMK1.1 derivative, MCS1: repeat-spacer-repeat fragment with *dmpG* spacer targeting coding strand		This study
pMK31	pMK1 derivative, MCS1: repeat-spacer-repeat fragment with *dmpG* spacer targeting non-coding strand; MCS2: *casdinG*		This study
pMK32	pMK1.1 derivative, MCS1: repeat-spacer-repeat fragment with *dmpG* spacer targeting non-coding strand		This study

pSR77	All-in-one Type IV CRISPR-Cas from *P.oleovorans*. pRFSDuet™-1 derivative, MCS1: (*cas6*, *cas8*, *cas7*, *cas5*); T7 promoter for repeat-spacer-repeat fragment with BseRI restriction site on the spacer; MCS2: *casdinG*		(Sanchez-Londono *et al.*, 2025)
pMSL88	pSR77 derivative. MCS2: *casdinG*+HNH domain of *Sulfitobacter sp.* JL08		This study
pMSL89	pMSL88 derivative with crRNA targeting *lacZ* non-coding strand		This study
pLCB1	pMSL88 derivative with crRNA targeting 2.6 kb downstream of BL21-AI *asp-tRNA*		This study

Supplementary Table S3: Oligonucleotides used for primer walking.

Name	Sequence (5′ → 3′)	Amplified region
oPS6	GCGTTCAATTTCGCGTGCCAGATG	*hemB*
oPS7	TTAATCCAACGCCCTCGTCGCC	*hemB*
oPS10	ACCTACCGCATTGGCAACCGTG	*lacY*
oPS12	GTCCCTGAGCTGGTGACGCAAC	*cynT*
oPS16	AGCGGCGGTGCACAATCTTCTC	*LacI*
oPS17	GCCACGCCATGTTTTGCTGGTG	*prpE*
oPS18	ACAGGCGGCTTTTCAGGTACGC	*prpR*
oPS19	GTGCAGGCTGGACGCTTTACCG	*yahL*
oPS20	AACCCCAACGTCACCATTCCGC	*yahG*
oPS21	CGTCATCGCCTCACGGACCATG	*yahB*
oPS23	TGTAACGCGCCTGTTCGAGCTG	*prpR*
oPS24	AAGCGACTGATGCGGTTGGTGG	*yahF*
oPS25	AACTTAATCGCCTTGCAGCACATC	*lacZ*
oPS26	CATCCGCCACATATCCTGATCTTCC	*lacZ*
oPS29	ACAAAGTCAGCGGTGAGCGTACC	*codB*
oPS30	GCATCACGTTGACGTTACCGATGC	*frmA*
oPS31	CGCTCACCATAACCTGAGTCTTGG	*dmpG*

oPS32	CGTTTGCCATACCGACGCATTTACC	*frmA*
oPS35	CTCTCTTTCGGAATGACAAACGCC	prpE
oPS38	CGCATTGATGGATAAGCTGCGTCC	*mhpT*
oPS41	TCGCAAGATAACAACTTTAGCCAGGG	*codB*
oPS43	CAAATCCCGCCAGCAACATACCG	*mhpT*
oPS44	AATACATCAGGCTCACTGCCGTCC	*mhpT*
oPS49	ACGGCCAGCGGATGATGTTGC	*cynR*
oPS50	ACTGATGCAGGAGAAGTCTGGCG	*cynR*
oPS51	AATCGGCATAACGCAGCCAGTGG	GSU80_01530
oPS54	GTGGATCCGGAAGCCTTTGGC	*dnaQ*
oPS55	GATAAACTTCCGCAAGGATCTGGGC	*dnaQ*
oPS57	GAGAACTCTGCATCCGCTCAACC	GSU80_01575
oPS58	ATCAGCACCAGTTTGCCGACGC	GSU80_01580
oPS61	TGCCTCAGATTCAGGTTATGCCGC	GSU80_01630
oPS62	ATGGACGAACAGTGGGGCTATGTC	GSU80_01630
oPS64	GTGCCTCGATTGTCGGCATCATG	*lacY*
MH88_oPS19_Rev	GCGAAGTGTCGCCGATCACAAC	*yahL*
MH89_oPS20_Rev	GCCACCAATGGCAACGACCACA	*yahG*
MH90_oPS24_For	GGCGATGGCGACCGAAATGAAC	*yahF*
MH91_oPS21_Rev	TGGCATTTACTACCGCTGCACG	*yahB*
MK88	CAACTTATTGATAGTGTTTTATG	IS1
MK101	AGAATTAGTCGTTATCGC	IS1
MK36	ATTTGCTGCCGGACAATTTC	GSU80_01415
MK37	CCTGCTTTCATTACCCATTC	GSU80_01416
LCB13	CGTCTTGATCTGGTGCAG	*tRNA-Asp gene*
MK116	CTACCATGCGGAACAGAT	*tRNA-Asp gene*

Supplementary Table S4: Protospacer sequences of genomic targets in the *E. coli* genome

Type IV-A1 CRISPR-Cas with CasDinG-HNH crRNA targeting *lacZ* non-coding strand	AGGCCCGCACCGATCGCCCTTCCCAACAGTTG
Type IV-A1 with delta NTD-CasDinG-HNH crRNA targeting *lacZ* coding strand	ATCGCACTCCAGCCAGCTTTCCGGCACCGCTT
Type I-Fv CRISPR-Cas with CasDinG and CasDinG mutant (K82A)	GGAAACCAGGCAAAGCGCCATTCGCCATTCAG
Type IV-A1 CRISPR-Cas with CasDinG-HNH crRNA targeting region close to tRNA-Asp	CTTATGCTTATTACATGCAAAACGTCCCGTTT
Type IV-A1 CRISPR-Cas with CasDinG-HNH or delta NTD-CasDinG-HNH crRNA non-targeting spacer (negative control)	ATGTGACTCTCCTCCGAAGAGGAGGTAAGTAC

Supplementary Table S5: WGS data analysis

Sample	Run Accession	Sample Accession	Secondary Sample Accession	Experiment Accession
Type- IV-CasDinG_HNH-LacZ (1st batch)	ERR15006951	SAMEA118348533	ERS24618778	ERX14411293
Type-IV-HNH_delta_NTD_LacZ (1st batch)	ERR15006952	SAMEA118348534	ERS24618779	ERX14411294
Type-IFV-CasDinG (1st batch)	ERR15006953	SAMEA118348535	ERS24618780	ERX14411295
Type-IFV-CasDinG_Mutant-K82A (1st batch)	ERR15006954	SAMEA118348536	ERS24618781	ERX14411296
Type-IV-WildType-LacZ	ERR15006955	SAMEA118348537	ERS24618782	ERX14411297
Type- IV-CasDinG_HNH-LacZ (2nd batch)	ERR15680354	SAMEA120308275	ERS26970262	ERX15084785
Type-IV-CasDinG-HNH-tRNA-Asp (2nd batch, new target)	ERR15680355	SAMEA120308276	ERS26970263	ERX15084786
Type-IFV-CasDinG_Mutant-K82A (2nd batch)	ERR15680356	SAMEA120308277	ERS26970264	ERX15084787
Type-IV-HNH_delta_NTD_LacZ (2nd batch)	ERR15680357	SAMEA120308278	ERS26970265	ERX15084788
Type-IFV-CasDinG (2nd batch)	ERR15680358	SAMEA120308279	ERS26970266	ERX15084789

Sample	Number of sequenced reads	Coverage Depth (×)	Mean coverage per genome (λ)	Probability that a genome is missed (P_{miss})	Expected number of genomes with zero reads
Type- IV-CasDinG_HNH-LacZ (1st batch)	16853336	871	4,36	1,284%	3

Type-IV-HNH_delta_NTD_LacZ (1st batch)	17411844	919	4,60	1,010%	2
Type-IFV-CasDinG (1st batch)	39911784	1414	7,07	0,085%	0
Type-IFV-CasDinG_Mutant-K82A (1st batch)	10534811	363	1,82	16,284%	33
Type-IV-WildType-LacZ	19479282	1055	5,28	0,512%	1
Type- IV-CasDinG_HNH-LacZ (2nd batch)	25333920	720	8,00	0,034%	0
Type-IV-CasDinG-HNH-tRNA-Asp (2nd batch, new target)	21857853	1312	14,58	0,000%	0
Type-IFV-CasDinG_Mutant-K82A (2nd batch)	26153958	311	3,46	3,157%	3
Type-IV-HNH_delta_NTD_LacZ (2nd batch)	34151316	923	10,26	0,004%	0
Type-IFV-CasDinG (2nd batch)	29300086	330	3,67	2,556%	2

Supplementary Figures

Supplementary Figure S1:

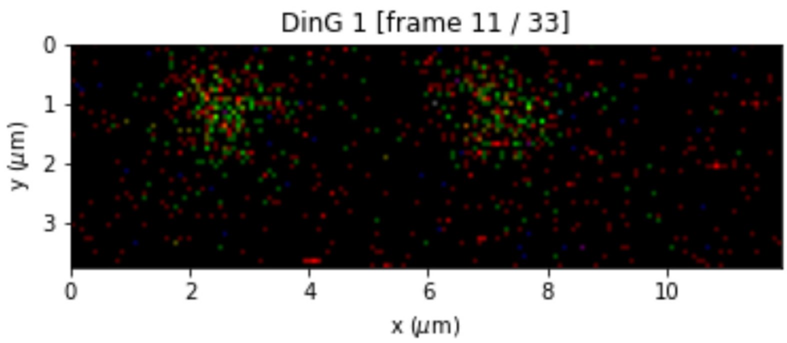

Supplementary Figure S1: C-trap measurement of labeled CasDinG in the absence of Type IV crRNPs. Screenshot of C-trap measurement of labeled CasDinG in the (i) absence of Type IV crRNPs and (ii) in presence of target DNA which is tethered between two optical traps (green dots). Recruitment of CasDinG was not observed.

Supplementary Figure S2:

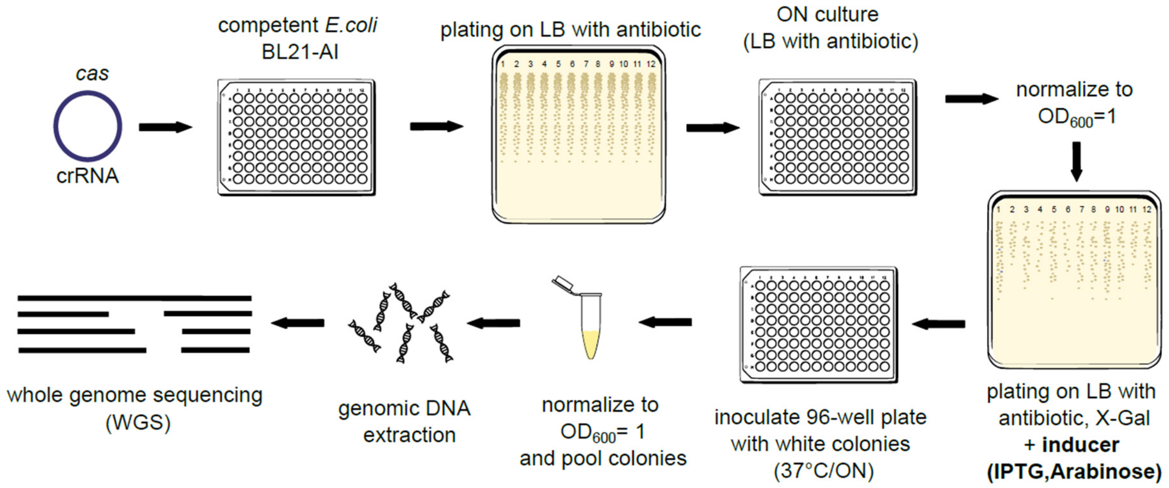

Figure S2: Workflow for genomic targeting assays with engineered type IV-A1 and type I-Fv CRISPR-Cas systems. Schematic of workflow to test the engineered variants of type IV-A1 and the type I-Fv system in *E. coli* BL21-AI cells by targeting *lacZ*. Constructs were transformed into small aliquots of competent cells (20 µl) and plated by dropping 15 µl on large square LB plates. ON cultures of these transformations were OD-normalized and plated on inducer plates. White colonies were inoculated, grown, OD-normalized and pooled for genomic DNA extraction. Genomic DNA was analyzed using whole genome sequencing.

Supplementary Figure S3:

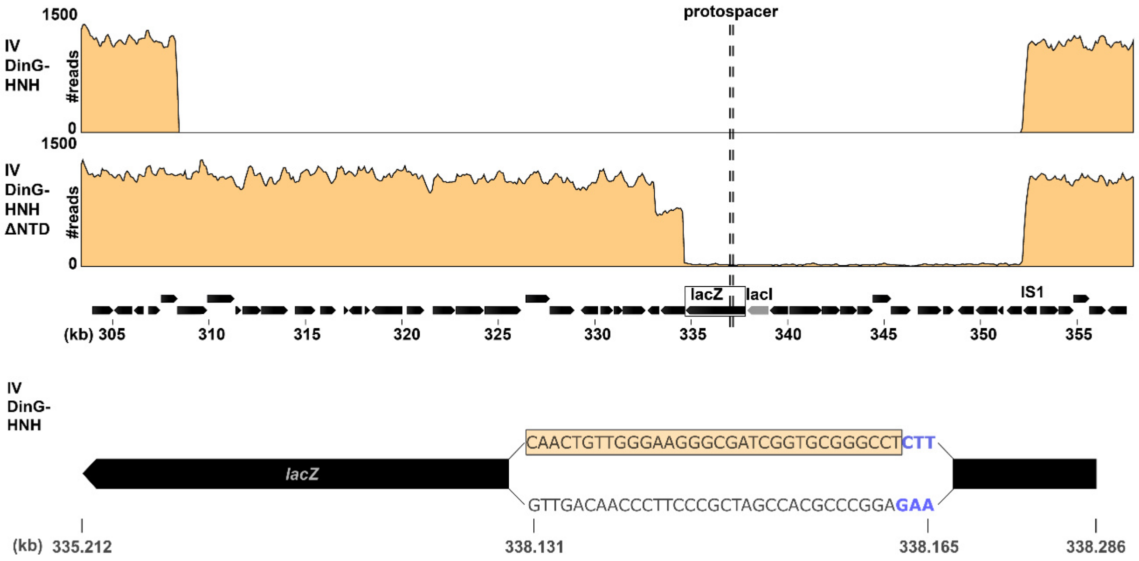

Figure *S3*: Whole genome sequencing analyses to identify deletions generated with engineered type IV-A systems. Top: Coverage plots of the neighboring region of the lacZ protospacer indicating the length of deletions generated with the engineered type IV-A1 systems. The type IV-A1 system with CasDinG-HNH generated a deletion of ~43.8 kb and the type IV-A1 system with CasDinGΔNTD deletions ranging from 17.6-34.1 kb. Middle: genomic organization shows the genomic position with the lacZ protospacer (dashed line). Reads of *lacI* gene have been excluded due to its presence in the expression vectors. Bottom: Close-up of the targeting region showing the protospacer (orange) and PAM (blue).

Supplementary Figure S4:

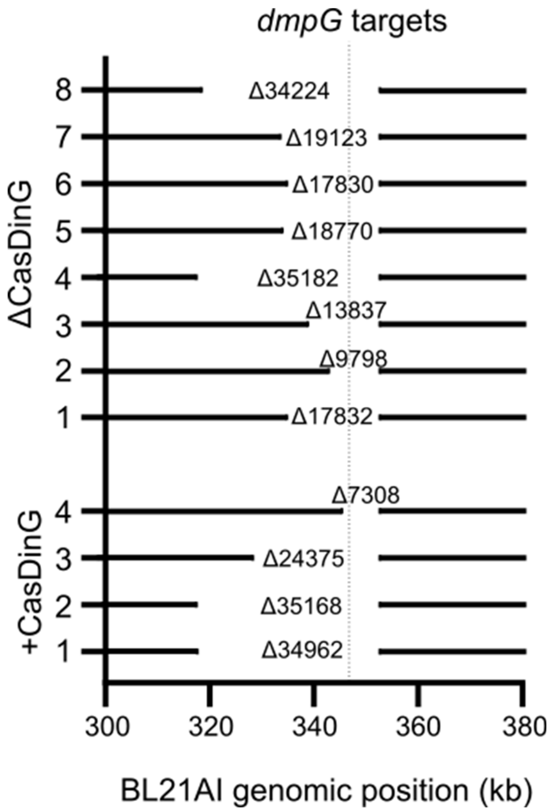

Supplementary Figure S4: Deletions of *E. coli* genomes with *dmpG* target. *E. coli* BL21 AI cells were assayed with type I-Fv with and without CasDinG and a spacer targeting either the coding or the non-coding strand of the gene *dmpG*. Different deletion sizes were observed using primer walking. All deletions share a conserved stopping site at an IRR sequence of an insertion sequence (IS1).

Supplementary Video S1

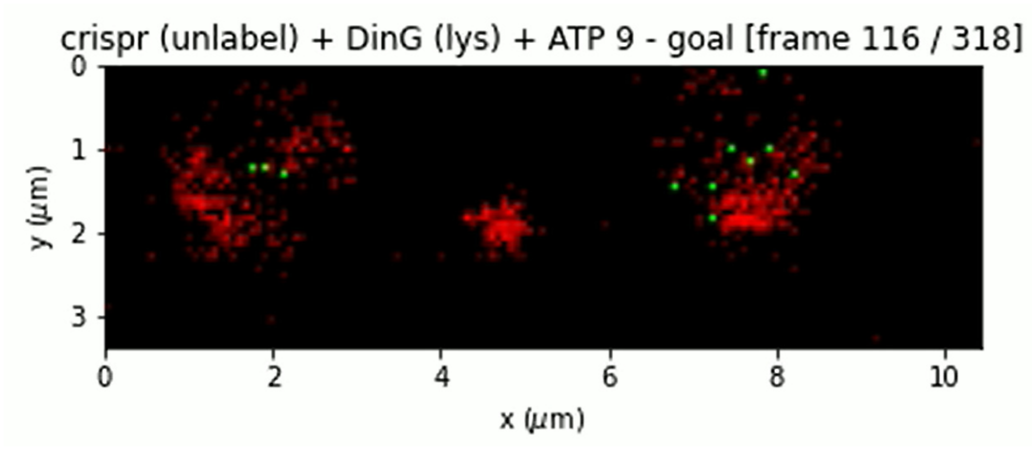

Confocal Visualization of CasDinG Recruitment to Target DNA. Time-lapse fluorescence microscopy video acquired using the C-Trap optical tweezers setup. CasDinG, labeled with a

red fluorophore, is shown binding to the DNA tether after recruitment by the type IV-A1 effector complex. Bead autofluorescence is shown in green. Video frames were analyzed to generate the histogram in Figure 1D.

Supplementary References

Čepaitė, R., Klein, N., Mikšys, A., Camara-Wilpert, S., Ragožius, V., Benz, F., Skorupskaitė, A., Becker, H., Žvejytė, G. and Steube, N. *et al.* (2024) Structural variation of types IV-A1- and IV-A3-mediated CRISPR interference, *Nat Commun,* **15,** 9306.

Gleditzsch, D., Müller-Esparza, H., Pausch, P., Sharma, K., Dwarakanath, S., Urlaub, H., Bange, G. and Randau, L. (2016) Modulating the Cascade architecture of a minimal Type I-F CRISPR-Cas system, *Nucleic Acids Research,* **44,** 5872–5882.

Guo, X., Sanchez-Londono, M., Gomes-Filho, J.V., Hernandez-Tamayo, R., Rust, S., Immelmann, L.M., Schäfer, P., Wiegel, J., Graumann, P.L. and Randau, L. (2022) Characterization of the self-targeting Type IV CRISPR interference system in Pseudomonas oleovorans, *Nature microbiology,* **7,** 1870–1878.

Rust, S. and Randau, L. (2025) Real-time imaging of bacterial colony growth dynamics for cells with Type IV-A1 CRISPR-Cas activity, *microLife,* **6,** uqaf006.
